# Supplementary material for: The anabolic response to protein ingestion during recovery from exercise has no upper limit in magnitude and duration in vivo in humans
Source: Cell Rep Med. 2023 Dec 19;4(12):101324. doi: 10.1016/j.xcrm.2023.101324 (PMC10772463; doi:10.1016/j.xcrm.2023.101324)
Supplement: Document S1. Figures S1 and S2 and Table S1 [file mmc1.pdf]

Cell Reports Medicine, Volume *4*

Supplemental information

The anabolic response to protein ingestion during recovery from exercise has no upper limit in magnitude and duration *in vivo* in humans

Jorn Trommelen, Glenn A.A. van Lieshout, Jean Nyakayiru, Andrew M. Holwerda, Joey S.J. Smeets, Floris K. Hendriks, Janneau M.X. van Kranenburg, Antoine H. Zorenc, Joan M. Senden, Joy P.B. Goessens, Annemie P. Gijsen, and Luc J.C. van Loon

SUPPLEMENTAL TITLES AND LEGENDS

Supplemental Figure 1. Plasma amino acid responses following protein ingestion. Related to figure 1.

Supplemental Figure 2. Muscle free amino acid response following protein ingestion. Related to figure 3.

Supplemental table 1. Subject’s characteristics. Related to figure 1.

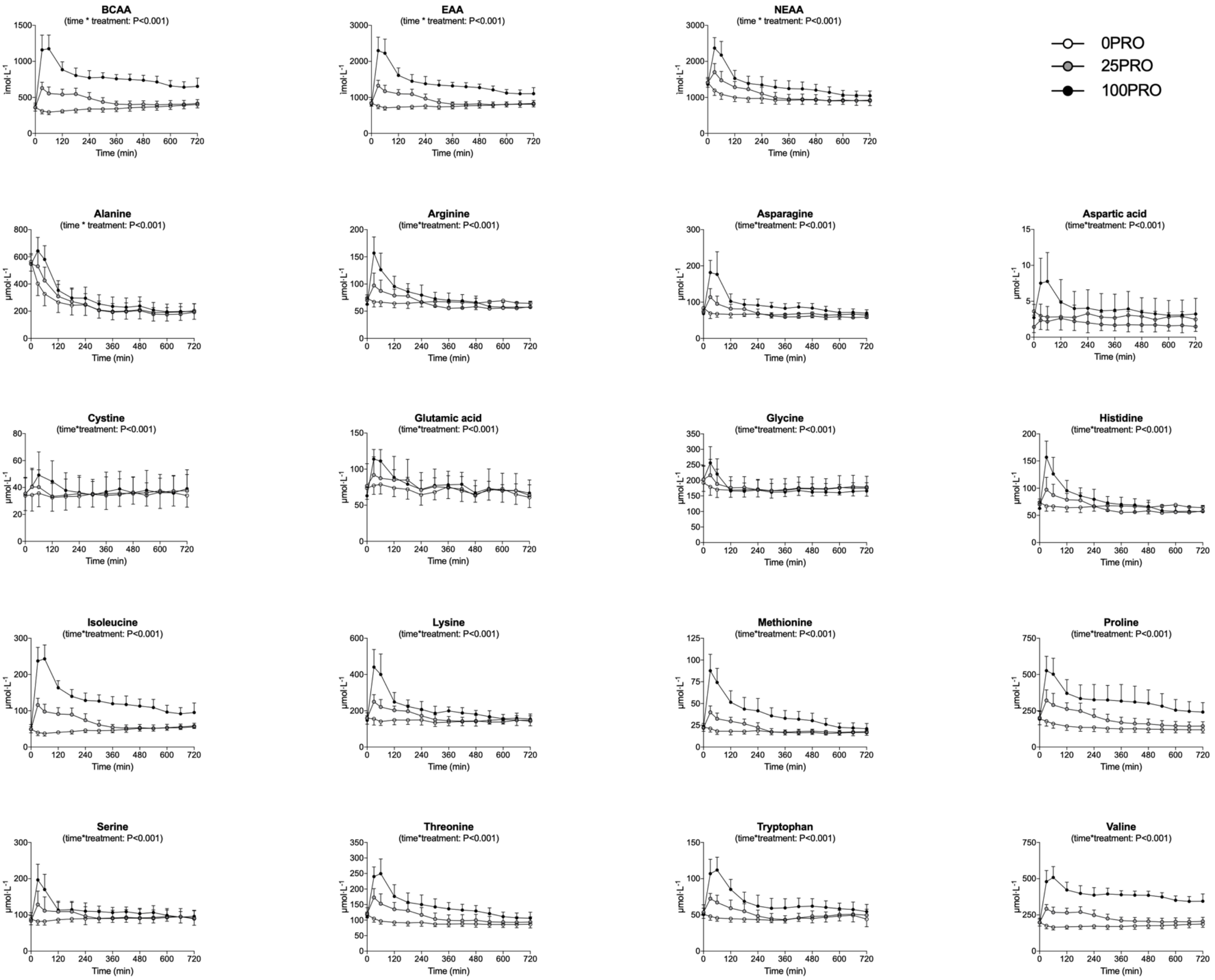

BCAA
(time * treatment: P<0.001)
EAA
(time * treatment: P<0.001)
NEAA
(time * treatment: P<0.001)
0PRO
25PRO
100PRO
Alanine
(time * treatment: P<0.001)
Arginine
(time*treatment: P<0.001)
Asparagine
(time*treatment: P<0.001)
Aspartic acid
(time*treatment: P<0.001)
Cystine
(time*treatment: P<0.001)
Glutamic acid
(time*treatment: P<0.001)
Glycine
(time*treatment: P<0.001)
Histidine
(time*treatment: P<0.001)
Isoleucine
(time*treatment: P<0.001)
Lysine
(time*treatment: P<0.001)
Methionine
(time*treatment: P<0.001)
Proline
(time*treatment: P<0.001)
Serine
(time*treatment: P<0.001)
Threonine
(time*treatment: P<0.001)
Tryptophan
(time*treatment: P<0.001)
Valine
(time*treatment: P<0.001)
µmol·L-1
Time (min)

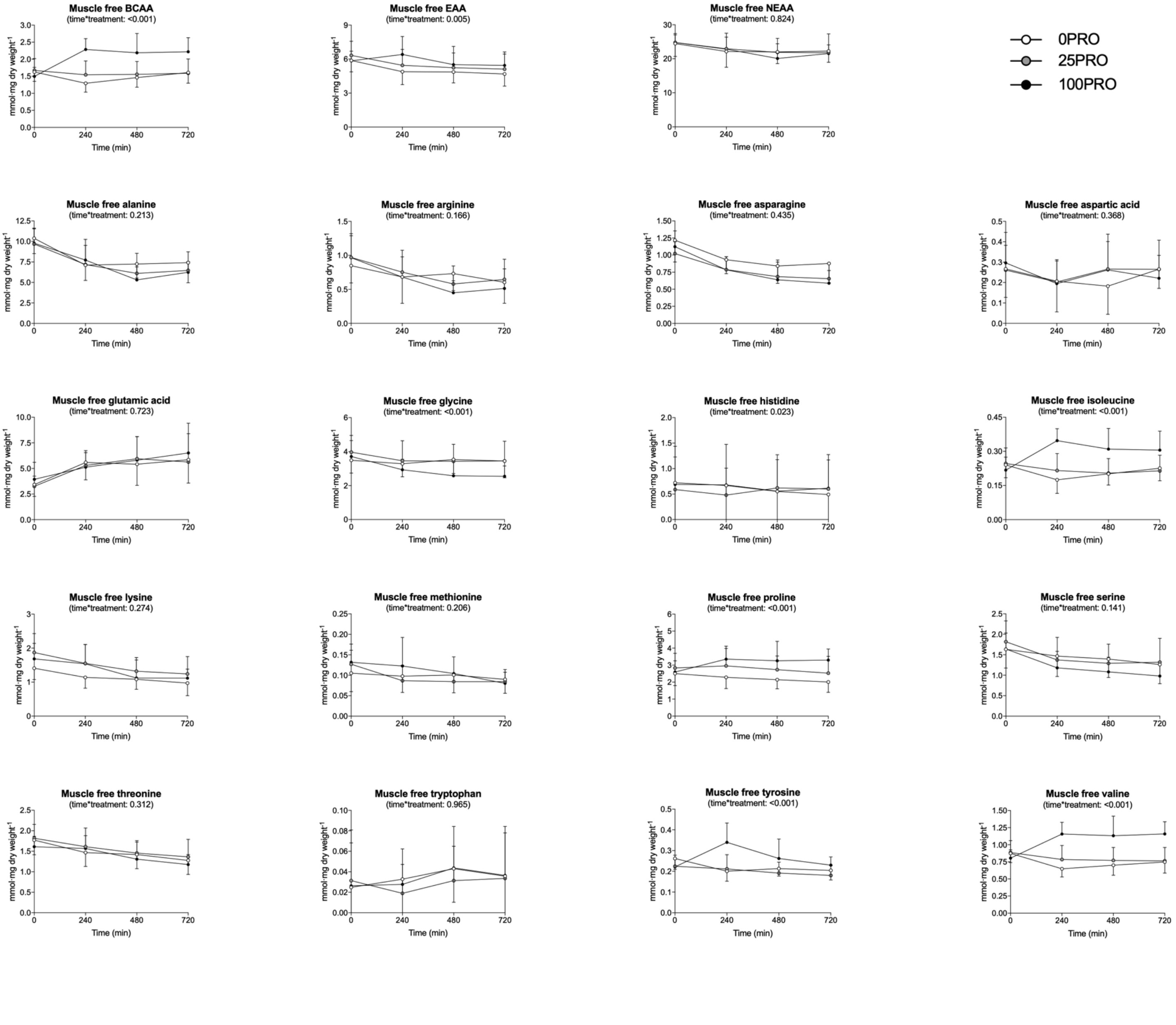
0PRO
25PRO
100PRO
Muscle free BCAA
(time*treatment: <0.001)
Muscle free EAA
(time*treatment: 0.005)
Muscle free NEAA
(time*treatment: 0.824)
Muscle free alanine
(time*treatment: 0.213)
Muscle free arginine
(time*treatment: 0.166)
Muscle free asparagine
(time*treatment: 0.435)
Muscle free aspartic acid
(time*treatment: 0.368)
Muscle free glutamic acid
(time*treatment: 0.723)
Muscle free glycine
(time*treatment: <0.001)
Muscle free histidine
(time*treatment: 0.023)
Muscle free isoleucine
(time*treatment: <0.001)
Muscle free lysine
(time*treatment: 0.274)
Muscle free methionine
(time*treatment: 0.206)
Muscle free proline
(time*treatment: <0.001)
Muscle free serine
(time*treatment: 0.141)
Muscle free threonine
(time*treatment: 0.312)
Muscle free tryptophan
(time*treatment: 0.965)
Muscle free tyrosine
(time*treatment: <0.001)
Muscle free valine
(time*treatment: <0.001)
mmol·mg dry weight-1
Time (min)

Supplemental table 1. Subject's characteristics

	0PRO (n=12)	25PRO (n=12)	100PRO (n=12)	Total (n=36)
Age (y)	24 ± 5	25 ± 5	27 ± 7	25 ± 6
Body mass (kg)	75.5 ± 14.1	79.1 ± 10.4	75.7 ± 10.6	76.8 ± 11.6
Height (m)	1.79 ± 0.08	1.83 ± 0.07	1.81 ± 0.08	1.81 ± 0.08
BMI (kg ·m^{-2})	23.5 ± 3.2	23.6 ± 3.2	23.0 ± 2.5	23.4 ± 2.9
Body fat (%)	17 ± 6	16 ± 6	14 ± 4	16 ± 5
Fat free mass (kg)	62.5 ± 7.7	66.3 ± 7.5	64.6 ± 8.1	64.5 ± 7.7
Skeletal muscle mass (kg)	32.3 ± 4.1	34.3 ± 4.4	33.4 ± 4.6	33.4 ± 4.3
1RM Leg Press (kg)	215 ± 61	197 ± 44	204 ± 39	205 ± 48
1RM Leg Extension (kg)	107 ± 28	108 ± 22	114 ± 24	110 ± 25
1RM Lat pulldown (kg)	80 ± 24	79 ± 14	79 ± 15	79 ± 18
1RM Chest press (kg)	89 ± 33	90 ± 23	93 ± 21	91 ± 25
Energy intake (MJ·kg BM^{-1}· d^{-1})	0.13 ± 0.02	0.12 ± 0.03	0.14 ± 0.03	0.13 ± 0.03
Protein intake (g·kg BM^{-1}· d^{-1})	1.4 ± 0.4	1.1 ± 0.3	1.5 ± 0.3	1.3 ± 0.3
Carbohydrate intake (g·kg BM^{-1}· d^{-1})	3.5 ± 0.9	3.5 ± 0.9	4.0 ± 1.3	3.7 ± 1.1
Fat intake (g·kg BM^{-1}· d^{-1})	1.1 ± 0.3	1.1 ± 0.3	1.2 ± 0.4	1.1 ± 0.3

Values represent mean ± SD. Dietary records data in PLA: n=11.
