# Supplementary material for: The anabolic response to protein ingestion during recovery from exercise has no upper limit in magnitude and duration in vivo in humans
Source: Cell Rep Med. 2023 Dec 19;4(12):101324. doi: 10.1016/j.xcrm.2023.101324 (PMC10772463; doi:10.1016/j.xcrm.2023.101324)
Supplement: Document S2. Article plus supplemental information [file mmc2.pdf]

Cell Reports
Medicine

Article

The anabolic response to protein ingestion during recovery from exercise has no upper limit in magnitude and duration *in vivo* in humans

Graphical abstract

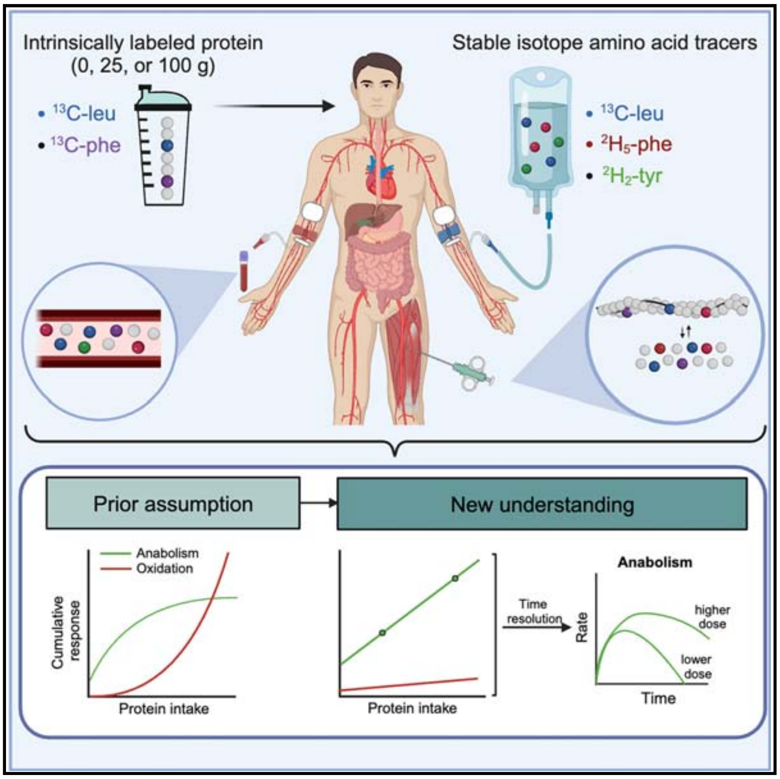

Highlights

- Postprandial protein anabolism remains elevated during prolonged hyperaminoacidemia
- Protein ingestion has a negligible impact on whole-body amino acid oxidation
- Muscle protein autophagy is not modulated by protein ingestion
- Exogenous amino acids are the main precursors in postprandial protein accretion

Authors

Jorn Trommelen, Glenn A.A. van Lieshout, Jean Nyakayiru, ..., Joy P.B. Goessens, Annemie P. Gijsen, Luc J.C. van Loon

Correspondence

l.vanloon@maastrichtuniversity.nl

In brief

Trommelen et al. investigate the anabolic response to the ingestion of various amounts of protein following exercise and demonstrate a dose-response increase in the magnitude and duration of protein absorption, muscle protein synthesis rates, and whole-body net protein balance. The anabolic response to protein ingestion has no upper limit.

Trommelen et al., 2023, Cell Reports Medicine *4*, 101324
December 19, 2023 © 2023 The Author(s).
https://doi.org/10.1016/j.xcrm.2023.101324

Cell Reports Medicine

CellPress
OPEN ACCESS

Article

The anabolic response to protein ingestion during recovery from exercise has no upper limit in magnitude and duration *in vivo* in humans

Jorn Trommelen,[1] Glenn A.A. van Lieshout,[1,2] Jean Nyakayiru,[1] Andrew M. Holwerda,[1] Joey S.J. Smeets,[1] Floris K. Hendriks,[1] Janneau M.X. van Kranenburg,[1] Antoine H. Zorenc,[1] Joan M. Senden,[1] Joy P.B. Goessens,[1] Annemie P. Gijsen,[1] and Luc J.C. van Loon[1,3,*]

[1]Department of Human Biology, NUTRIM School of Nutrition and Translational Research in Metabolism, Maastricht University Medical Centre+, Maastricht, the Netherlands
[2]FrieslandCampina, 3818 LE Amersfoort, the Netherlands
[3]Lead contact
*Correspondence: l.vanloon@maastrichtuniversity.nl
https://doi.org/10.1016/j.xcrm.2023.101324

SUMMARY

The belief that the anabolic response to feeding during postexercise recovery is transient and has an upper limit and that excess amino acids are being oxidized lacks scientific proof. Using a comprehensive quadruple isotope tracer feeding-infusion approach, we show that the ingestion of 100 g protein results in a greater and more prolonged (>12 h) anabolic response when compared to the ingestion of 25 g protein. We demonstrate a dose-response increase in dietary-protein-derived plasma amino acid availability and subsequent incorporation into muscle protein. Ingestion of a large bolus of protein further increases whole-body protein net balance, mixed-muscle, myofibrillar, muscle connective, and plasma protein synthesis rates. Protein ingestion has a negligible impact on whole-body protein breakdown rates or amino acid oxidation rates. These findings demonstrate that the magnitude and duration of the anabolic response to protein ingestion is not restricted and has previously been underestimated *in vivo* in humans.

INTRODUCTION

Skeletal muscle tissue is in a constant state of turnover, with coordinated up- and down-regulation of muscle protein synthesis and muscle protein breakdown rates. This turnover allows muscle tissue to remodel by replacing damaged proteins and changing the muscle tissue protein composition. A net gain or loss of muscle protein occurs as a consequence of a positive or negative muscle protein balance (synthesis minus breakdown). Because muscle protein synthesis rates have a higher capacity to fluctuate when compared to muscle protein breakdown rates, postprandial muscle protein balance is assumed to be largely governed by changes in muscle protein synthesis rates.[1,2] Increases in muscle protein synthesis rates are mainly driven by protein ingestion and muscle contraction.[1–5]

Stable isotope methodology allows the assessment of dietary-protein-derived amino acid kinetics and the metabolic fate of ingested-protein-derived amino acids.[6] Briefly, ingested protein is first digested, after which amino acids are absorbed in the gut.[7–9] Some of the absorbed amino acids undergo first-pass splanchnic extraction,[10,11] but the majority of the protein-derived amino acids are released into the circulation, where they can be taken up by the peripheral tissues.[4,9,12] Subsequently, the amino acids can either be incorporated into tissue protein (protein synthesis) and/or catabolized (oxidation).[9,13] Protein-derived amino acids not only serve as metabolic precursors but also act as important signaling molecules that regulate anabolic and catabolic pathways.[14–16] Specifically, the mammalian target of rapamycin complex 1 (mTOR1) signaling cascade, which regulates cell growth and metabolism, is particularly sensitive to changes in plasma essential amino acid and leucine availability.[17]

Dose-response studies have reported that ingestion of 20–25 g protein is sufficient to maximize postexercise muscle protein synthesis rates in healthy, young adults, with no further increase in muscle protein synthesis rates when larger amounts of protein are consumed.[18–20] Higher protein intake did not further increase anabolic signaling, and excess amino acids are suggested to be oxidized.[18,19] Consequently, it is currently advised to distribute protein intake evenly throughout the day, with each main meal providing no more than 20–25 g protein.[21,22] Though this makes sense based on typical dietary intake patterns in humans, it seems to be at odds with the feeding practices of many animal species in nature that consume large amounts of food infrequently.[23] This is most notable in snakes, in which the ingestion of a large meal (≥25% body mass [BM]) results in prolonged protein digestion, amino acid absorption, and elevated protein synthesis rates (~10 days), with only ~5% of the ingested protein being directed toward amino acid oxidation.[24–27] Such data challenge the concept of transient anabolic responses to feeding in humans and the concept of muscle tissue having a limited

© 2023 The Author(s).
This is an open access article under the CC BY license (http://creativecommons.org/licenses/by/4.0/).

capacity to incorporate dietary-derived amino acids. Furthermore, this concept has been based on metabolic tracer studies *in vivo* in humans that have been confined to the assessment of the postprandial protein synthetic response following the ingestion of moderate amounts of protein ($\leq$45 g) over relatively short durations ($\leq$6 h).[18–20] When ingesting larger amounts of protein, such a short time frame will likely be insufficient to allow complete digestion and amino acid absorption[12] and, therefore, does not provide a reliable assessment of postprandial protein handling.

In the present study, we comprehensively assessed the time resolution of postprandial protein handling in response to the ingestion of moderate and large amounts of protein (0, 25, and 100 g) following exercise on a whole-body, muscle-tissue, and myocellular level *in vivo* in humans. To this end, we applied a quadruple isotope tracer feeding-infusion approach combined with frequent plasma and muscle tissue sample collection over a 12-h period. Here, we confirm our hypothesis of a dose-dependent increase in the magnitude and duration of protein-derived amino acid availability and muscle and whole-body protein synthesis rates, with a negligible impact on amino acid oxidation. This work highlights that tissues have a much higher capacity to incorporate exogenous-protein-derived amino acids than previously assumed and that the duration of the postprandial period is proportional to the size of the ingested meal.

RESULTS

To test our hypothesis, we produced intrinsically labeled milk protein, which was subsequently applied in a randomized controlled trial in human participants. Subjects were randomized to receive 25 g protein (25PRO), 100 g protein (100PRO), or a placebo (0PRO) following a single bout of resistance-type exercise. Stable isotope amino acid infusions were applied, and blood and muscle tissue samples were collected over time to assess the time course of postprandial protein handling. See Figure 1A for schematics of the study design. A detailed description of the methods applied is presented in the STAR Methods section.

Production of tailored intrinsically labeled protein to avoid destabilizing precursor enrichments

Here, we improved on the production method of intrinsically labeled protein to maintain steady-state tracer conditions following protein ingestion.[28] In brief, we produced higher and lower labeled protein batches to fine-tune the final enrichment level. We calculated that an intrinsically L-[1-^{13}C]-leucine labeled protein enrichment of 8 MPE would be required to maintain a steady state in plasma L-[1-^{13}C]-leucine enrichment following protein ingestion. However, the production of milk protein with a tailored enrichment level has remained difficult due to biological variation in cows and their milk yield. To circumvent this issue, we produced higher and lower enriched milk protein concentrate (MPC) batches by collecting milk both during and following the cow tracer infusion period, respectively. L-[1-^{13}C]-leucine enrichment was 10.8 and 2.4 MPE in the higher and lower enriched MPC batches, respectively. L-[1-^{13}C]-phenylalanine enrichment was 38.3 and 7.7 MPE in the higher and lower enriched MPC batches, respectively. Accounting for the amino acid profile and enrichment level of both batches, we mixed the higher and lower enriched MPCs in a 67:33 ratio to produce MPC with an L-[1-^{13}C]-phenylalanine enrichment of 31.6 MPE and an L-[1-^{13}C]-leucine enrichment of 8.0 MPE. Thus, we demonstrate here for the first time that the production of higher and lower intrinsically labeled protein batches from the same animal experiment allows subsequent mixing to ensure that the desired enrichment level is achieved.

Sustained hyperaminoacidemia following the ingestion of a large amount of protein

The plasma amino acid response following protein ingestion is often used as a proxy for protein digestion, amino acid absorption, and overall protein bioavailability.[7] Furthermore, increases in circulating amino acid concentrations represent major determinants of the muscle and whole-body protein synthetic response to feeding.[29,30] We applied frequent blood sampling to assess the time course of the plasma amino acid response to protein feeding. We observed elevated plasma alanine concentrations immediately following exercise and before protein consumption (t = 0 min; Figure S1), indicating that the resistance-type exercise bout elicited an acute stress response.[31]

Protein ingestion rapidly increased plasma amino acid concentrations in a dose-dependent manner (Figure 1D). Plasma amino acid concentrations were higher during the first 5 h following ingestion of 25 g protein when compared to the control condition (placebo). Ingestion of 100 g protein resulted in a greater rise in circulating plasma amino acid concentrations, which remained higher throughout the entire 12-h postprandial period. In line with this, the postprandial increase in plasma L-[1-^{13}C]-phenylalanine enrichments (tracer originating exclusively from the ingested protein) was greater and more prolonged following ingestion of 100 compared to 25 g protein (Figure 1J). Plasma L-[$^{2}H_5$]-phenylalanine enrichments (tracer originating exclusively from the intravenous infusion) showed a dose-response deviation from the steady-state conditions (Figure 1H). In line with our calculations, plasma L-[1-^{13}C]-leucine enrichments (tracer originating from both the ingested protein and the intravenous infusion) were ~8 MPE before protein ingestion and remained completely stable throughout the postprandial period regardless of the amount of ingested protein (Figure 1I).

Our data demonstrate that the magnitude and duration of the plasma amino acid response increases with a greater dose of ingested protein. Furthermore, a combination of stable isotope infusion and ingestion of specifically tailored intrinsically labeled protein is required to maintain postprandial plasma tracer steady-state conditions following the ingestion of a single bolus of protein.

Dose-dependent increases in amino acid kinetics and whole-body protein metabolism

Plasma amino acid concentrations and amino acid tracer enrichments are the net consequence of multiple amino acid fluxes into and out of the systemic circulation. Therefore, we applied a quadruple isotope tracer feeding-infusion method to quantify both exogenous and endogenous plasma amino acid kinetics. Exogenous-dietary-protein-derived amino acid appearance into the circulation was significantly higher in 100PRO when compared to 25PRO over the entire 12-h postprandial period

CellPress
OPEN ACCESS

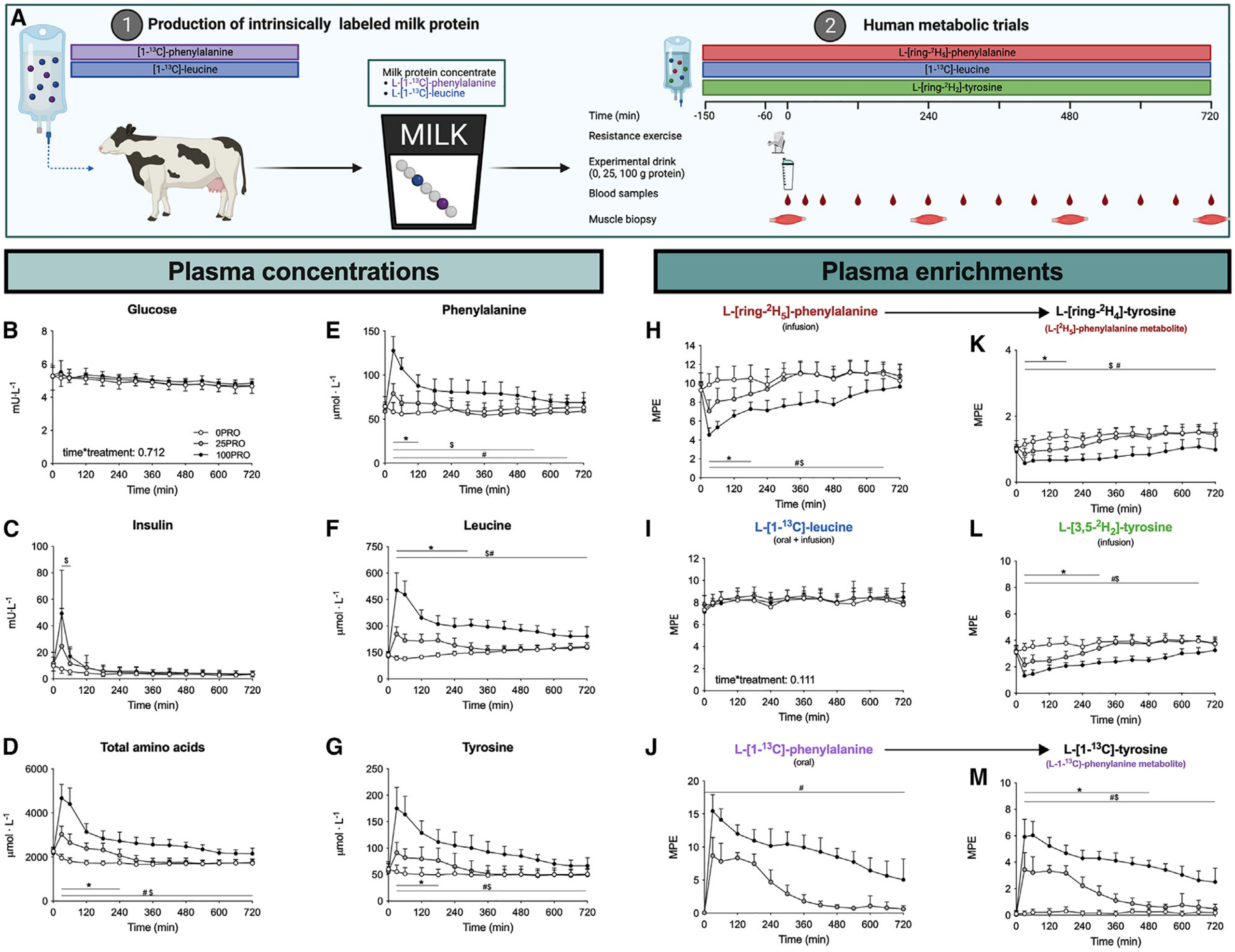

Figure 1. Schematics of study design and plasma amino responses following protein ingestion

(A) Experimental scheme. Production of intrinsically labeled protein followed by human tracer study in which subjects ingested 0, 25, or 100 g protein in a single bolus (0PRO, 25PRO, and 100PRO, respectively).

(B–G) Plasma glucose (B), insulin (C), total amino acid (D), phenylalanine (E), leucine (F), and tyrosine (G) concentrations per treatment following test drink ingestion.

(H–M) Plasma L-[ring-$^{2}H_5$]-phenylalanine (H), L-[1-^{13}C]-leucine (I), L-1-[1-^{13}C]-phenylalanine (J), L-[ring-$^{2}H_4$]-tyrosine (K), L-[3,5-$^{2}H_2$]-tyrosine (L), and L-[1-^{13}C]-tyrosine (M) enrichments.

Unless otherwise stated, time-dependent data were analyzed with a two-factor repeated-measures ANOVA with time as a within-subjects factor and treatment group as a between-subjects factor. Bonferroni post hoc analysis was performed whenever a significant F ratio was found to isolate specific differences. All values are means + SD. Statistical significance was set at $p < 0.05$. *: 25PRO significantly different from 0PRO; $: 100PRO significantly different from 0PRO; #: 100PRO significantly different from 25PRO. For all measurements, n = 12 biological replicates. Figure S1 displays the graphs of other individual amino acids. See also Figure S1 and Table S1.

(Figure 2B). In contrast, the endogenous tissue protein-derived amino acid rates of appearance into the circulation did not differ between treatments (Figure 2C). The total rate of amino acid disappearance from the circulation into peripheral tissues increased in a dose-dependent manner (Figure 2D), with no evidence of any saturation effect.

The assessment of exogenous and endogenous plasma amino acid kinetics allowed us to calculate whole-body protein metabolism. It is generally believed that protein ingestion stimulates whole-body protein synthesis rates up to a maximal level, after which excess amino acids are being directed toward oxidation.[13,32] Here, we observed a further increase in both whole-body protein synthesis and amino acid oxidation rates following the ingestion of 25 and 100 g protein, respectively (Figures 2G and 2H). However, the concurrent increase in amino acid oxidation rates was negligible when compared to the whole-body protein synthetic response (Figure 2J). Collectively, this resulted in a strong positive correlation between protein intake and whole-body protein net balance (Figure 2L). These data demonstrate that the ingestion of a large amount of protein results in prolonged protein digestion, amino acid absorption, and continued amino acid release into the circulation. Subsequently,

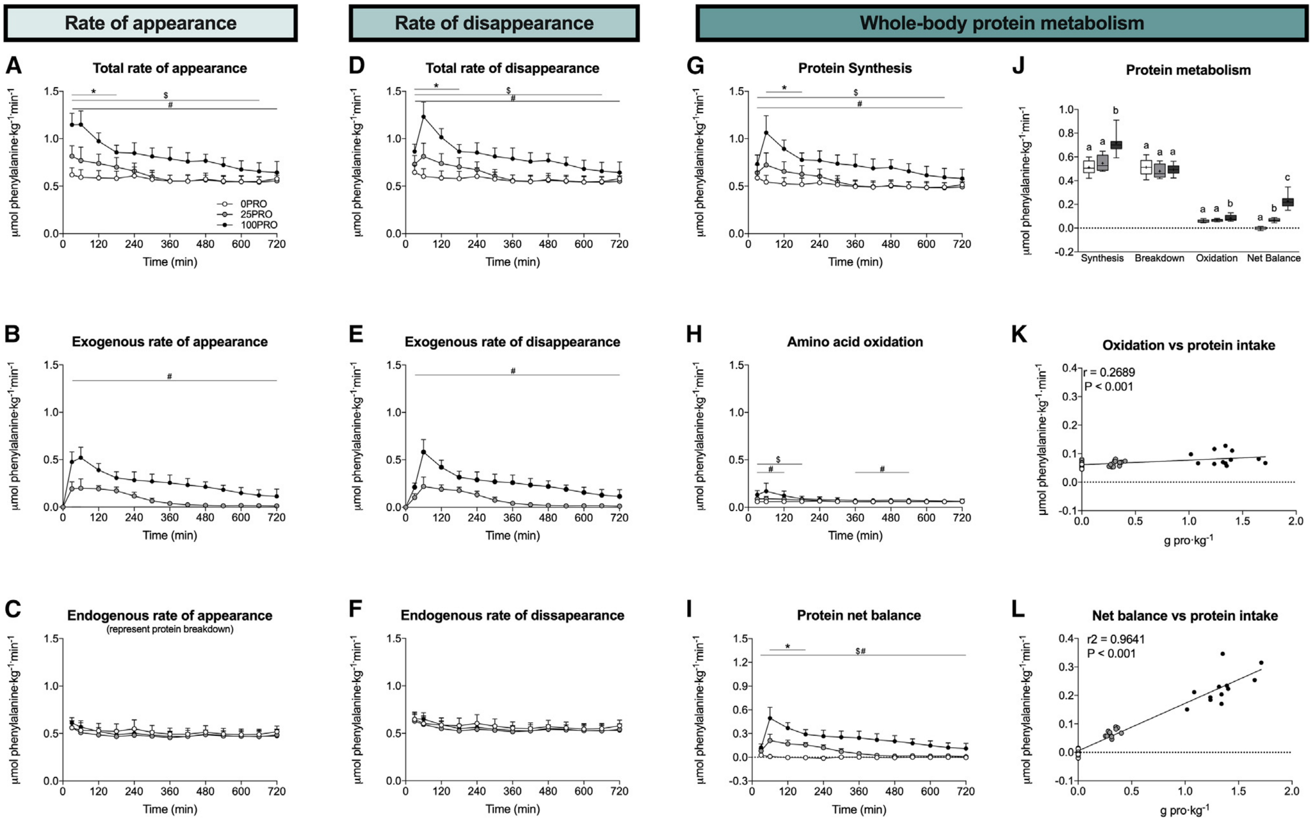

Figure 2. Dose-dependent increases in plasma amino acid kinetics and whole-body protein metabolism following protein ingestion

(A–C) Total (A), exogenous (B), and endogenous (C) rates of phenylalanine appearance.

(D–F) Total (D), exogenous (E), and endogenous (F) rates of phenylalanine disappearance.

(G–I) Whole-body protein synthesis (G), amino acid oxidation (H), and protein net balance (I) rates.

(J) Average whole-body metabolism rates.

(K) Correlation analysis of whole-body amino acid oxidation and protein intake relative to body mass.

(L) Correlation analysis of whole-body protein net balance and protein intake relative to body mass.

Unless otherwise stated, non-time-dependent data were analyzed with a one-way ANOVA with treatment group as a between-subjects factor. Bonferroni post hoc analysis were performed whenever a significant F ratio was found to isolate specific differences. (A–I) All values are means ± SD. Data in box and whiskers include the median (line), mean (cross), interquartile range (box), and minimum and maximum values (tails). Treatments without a common letter per time interval are significantly different. For all measurements, n = 12 biological replicates.

amino acids are taken up into tissues, are incorporated into tissue protein, and increase whole-body protein balance proportional to the ingested amount of protein.

Expansion of the tissue free branched-chain amino acid pool

The assessment of whole-body protein metabolism assumes that amino acids taken up from the circulation are either incorporated into tissues (protein synthesis) or catabolized (amino acid oxidation). However, amino acids taken up by tissues may also temporarily increase the tissue free amino acid pool.[33] Therefore, we assessed tissue free amino acid concentrations in muscle. Muscle tissue free phenylalanine concentrations (the amino acid used for the assessment of whole-body amino acid metabolism) did not differ between treatments (Figure 3A). These data imply that the previously observed postprandial expansion of the muscle free phenylalanine pool is short lived (<4 h), which will only impact the assessment of whole-body protein metabolism when it is assessed over shorter postprandial periods. In contrast to the clear dose-response increase in plasma total amino acid concentrations, muscle tissue free amino acid concentrations were not impacted by protein ingestion (Figure 3J). In fact, muscle tissue free total amino acid concentrations showed a minor decline over time. An exception to this pattern were the branched-chain amino acids leucine (Figure 3B), isoleucine, and valine, which were significantly higher in 100PRO over the entire 12-h postprandial period when compared to 25PRO or 0PRO (Figure S2). Muscle tissue free L-[$^{2}H_{5}$]-phenylalanine (Figure 3D) and L-[1-^{13}C]-leucine (Figure 3E) enrichments displayed steady-state conditions in all treatments. In contrast, muscle free L-[1-^{13}C]-phenylalanine enrichments showed a dose- and time-dependent increase following protein ingestion (Figure 3F). We observed an increase in muscle-protein-bound L-[1-^{13}C]-phenylalanine enrichments following protein ingestion, demonstrating that dietary-protein-derived amino acids are incorporated into muscle protein (Figures 3G–3I). Muscle-protein-bound L-[1-^{13}C]-phenylalanine

CellPress
OPEN ACCESS

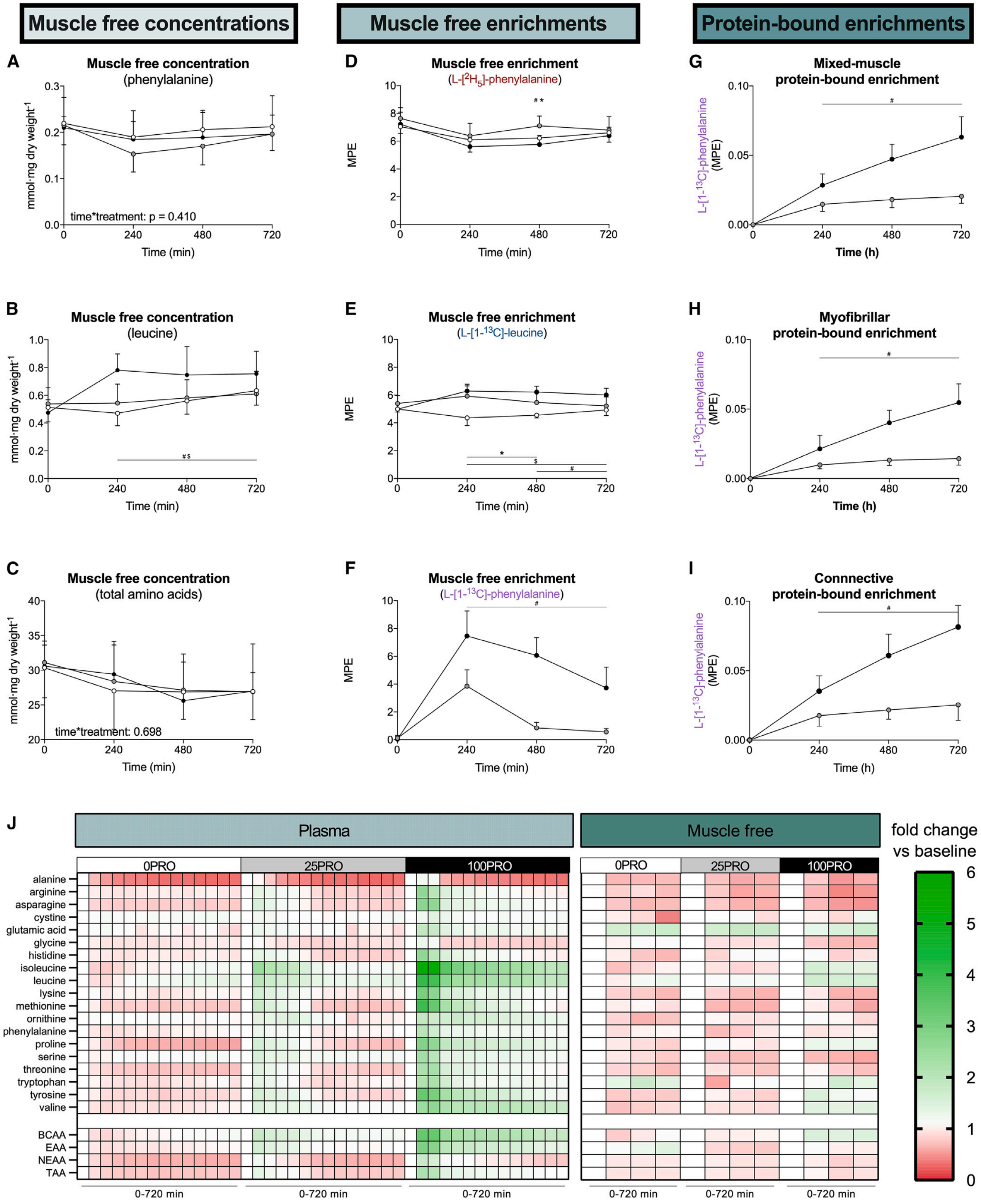

(legend on next page)

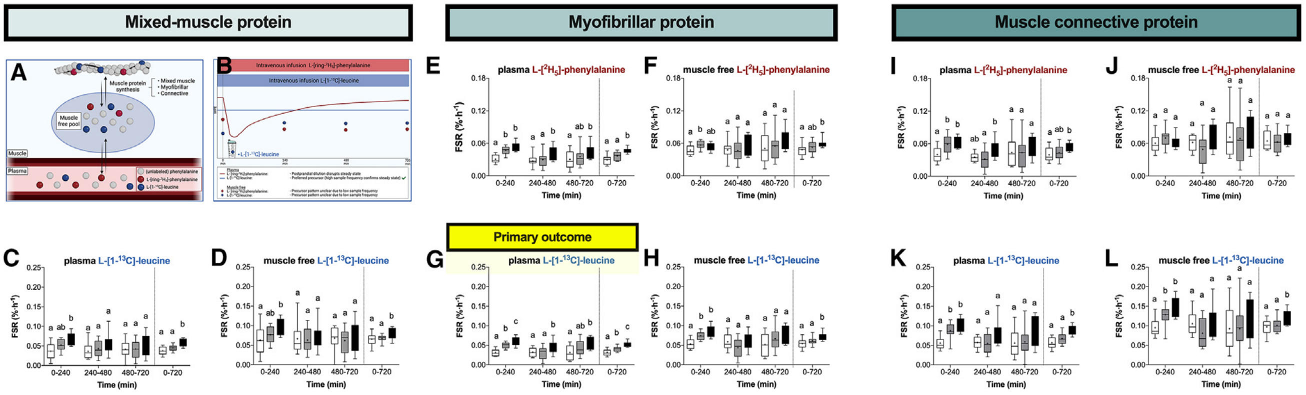

Figure 4. Prolonged increase in muscle protein synthesis rates following ingestion of a large amount of protein
(A) Scheme demonstrating the various amino acid tracers, precursor pools, and muscle protein fractions.
(B) Scheme demonstrating the expected time resolution of the various amino acid tracers and precursor pools.
(C) Mixed-muscle protein synthesis (MPS) rates based on L-[1-^{13}C]-leucine and the plasma precursor pool.
(D) MPS rates based on L-[1-^{13}C]-leucine and the muscle free precursor pool.
(E) Myofibrillar protein synthesis (MyoPS) based on L-[$^2H_5$]-phenylalanine and the plasma precursor pool.
(F) MyoPS based on L-[$^2H_5$]-phenylalanine and the muscle free precursor pool.
(G) MyoPS based on L-[1-^{13}C]-leucine and the plasma precursor pool (primary study outcome).
(H) MyoPS based on L-[1-^{13}C]-leucine and the muscle free precursor pool.
(I) Muscle connective protein synthesis (ConnectivePS) based on L-[$^2H_5$]-phenylalanine and the plasma precursor pool.
(J) ConnectivePS based on L-[$^2H_5$]-phenylalanine and the muscle free precursor pool.
(K) ConnectivePS based on L-[1-^{13}C]-leucine and the plasma precursor pool.
(L) ConnectivePS based on L-[1-^{13}C]-leucine and the muscle free precursor pool.
Data in box and whiskers include the median (line), mean (cross), interquartile range (box), and minimum and maximum values (tails). For all measurements, n = 12 biological replicates.

enrichments were increased in the first 4 h following the ingestion of 25 g protein, with little further increase during the subsequent 8-h period. In contrast, muscle-protein-bound L-[1-^{13}C]-phenylalanine incorporation increased linearly throughout the entire 12-h postprandial period following the ingestion of 100 g protein.

Our data confirm the assumption of a stable muscle free pool that is required for the accurate assessment of whole-body protein metabolism. Furthermore, we demonstrate for the first time a selective increase in leucine availability in the muscle free pool, which may support its role as a signaling molecule. In addition, these data confirm our hypothesis that the ingestion of a large amount of protein results in a prolonged and greater dietary-protein-derived amino acid incorporation into muscle tissue.

Prolonged muscle protein synthetic response to protein ingestion

Having established an increase in (exogenous) dietary-protein-derived amino acid incorporation into muscle tissue, we wanted to determine whether this translated to greater postprandial muscle protein synthesis rates. We assessed the muscle protein synthetic response in multiple protein fractions (mixed-muscle, myofibrillar protein, and muscle connective protein), based on two precursor pools (plasma and muscle free enrichments), with two different amino acid tracers (L-[$^2H_5$]-phenylalanine and L-[1-^{13}C]-leucine) over 4-h intervals during the 12-h postprandial period (Figures 4A and 4B). Myofibrillar protein synthesis rates over the entire 12-h postprandial period using the plasma L-[1-^{13}C]-leucine precursor pool were the predefined primary outcome, as frequent plasma sampling allows confirmation that our combined isotope tracer feeding-infusion method successfully maintained L-[1-^{13}C]-leucine steady-state conditions despite the ingestion of different amounts of protein.

In agreement with our hypothesis, we observed a clear dose-dependent pattern in muscle protein synthesis rates when assessed over the entire 12-h postprandial period (100PRO > 25PRO > 0PRO). Only myofibrillar protein synthesis rates based on the plasma L-[1-^{13}C]-leucine precursor differed significantly between all 3 treatments (Figure 4G). We expected that the greater cumulative muscle protein synthetic response in 100PRO vs. 25PRO would be predominately the result of a more sustained anabolic response of the myofibrillar protein

Figure 3. Changes in muscle free amino acid concentrations, enrichments, and exogenous amino acid incorporation into skeletal muscle
(A–C) Muscle free phenylalanine (A), leucine (B), and total amino acid (C) concentrations.
(D–F) Muscle free L-[$^2H_5$]-phenylalanine (D), L-[1-^{13}C]-leucine (E), and (L-[1-^{13}C]-phenylalanine) (F) enrichments.
(G–I) Protein-bound L-[1-^{13}C]-phenylalanine enrichments in mixed-muscle (G), myofibrillar (H), and muscle connective (I) protein.
(J) Heatmap of plasma and muscle free amino acid concentration fold changes per treatment following test drink ingestion compared to baseline.
All values are means ± SD. For all measurements, n = 12 biological replicates.
See also Figure S2.

CellPress
OPEN ACCESS

fraction. In line with this, myofibrillar protein synthesis rates were only ∼20% higher during the early 0- to 4-h period but ∼40% higher during the subsequent 4- to 12- h period in 100PRO when compared to 25 PRO.

Previous studies have failed to observe an anabolic effect of protein ingestion on muscle connective protein synthesis rates.[34–36] Here, we observed higher muscle connective protein synthesis rates following ingestion of 25 and 100 g protein throughout the initial 4-h postprandial period (Figure 4K). In addition, muscle connective protein synthesis rates were also higher following ingestion of 100 g protein when compared to the placebo condition over the entire 12-h postprandial period, with intermediate values following ingestion of 25 g protein. This anabolic response was evident despite the absence of an increase in plasma or muscle tissue free glycine concentrations (Figures 3J and S2). In fact, muscle free glycine concentrations were significantly decreased following the ingestion of 100 g protein. In contrast to our prior speculations,[34,35] these data indicate that elevated postprandial glycine concentrations in the plasma and muscle tissue free pool are not essential to allow a postprandial increase in muscle connective protein synthesis rates. Overall, both the initial increase and the duration of the postprandial increase in muscle protein synthesis rates are largely dependent on the amount of protein ingested.

Dissociation between postprandial muscle physiological and molecular responses

It has been well established that protein ingestion results in the upregulation of anabolic signaling and amino acid transport gene expression.[15,16] To gain more insight into the regulation of the muscle protein synthetic response to protein ingestion, we assessed the protein signaling and gene expression levels of selected targets involved in muscle anabolism, catabolism, and amino acid uptake. We observed time-dependent changes in all the selected genes known to be upregulated following an exercise bout. In contrast, we did not observe any impact of protein feeding on muscle protein signaling or muscle gene expression (Figure 5). This is in line with previous work that suggests that the molecular response in skeletal muscle to protein ingestion is short lived (<4 h).[16,37] Here, we extend these data by demonstrating that the muscle anabolic response to protein ingestion is sustained well beyond (≥12 h) the frequently reported transient changes in molecular signaling. Collectively, these data suggest substantial divergence in the time course of the molecular response and the actual metabolic response to feeding (Figure 5Y). Therefore, the magnitude and/or time course of muscle molecular responses should not be used as a proxy to evaluate the magnitude of the postprandial anabolic response to feeding.

No limit to protein-derived amino acid bioavailability and subsequent tissue incorporation

The production of intrinsically labeled protein allowed us to quantify the metabolic fate of ingested-protein-derived amino acids. The absolute amount of dietary-protein-derived amino acids appearing in the circulation over the 12-h postprandial period was substantially higher following ingestion of 100 when compared to 25 g protein (53 ± 7 vs. 16 ± 1 g, respectively) (Figures 6A and 6D). When expressed as a percentage of the ingested protein, cumulative dietary-protein-derived amino acid release into the circulation averaged 51%, 62%, and 66% over 4, 8, and 12 h, respectively, following the ingestion of 25 g protein. In contrast, dietary-protein-derived amino acid release into the circulation following ingestion of 100 g protein did not plateau, with 26%, 44%, and 53% of the ingested-protein-derived amino acids appearing over 4, 8, and 12 h, respectively. These data show that the ingestion of larger amounts of protein requires a more prolonged period to allow full digestion, amino acid absorption, and subsequent amino acid release into the circulation.

We have previously estimated that the ingestion of 20 g protein results in ∼10% (∼2 g) of the ingested-protein-derived amino acids to become incorporated in skeletal muscle tissue throughout a 6-h postprandial period.[4] In the current study, we calculated that 12%, 15%, and 18% of the ingested protein was incorporated into skeletal muscle following ingestion of 25 g protein throughout 4-, 8-, and 12-h periods, respectively (Figures 6B and 6D). The higher values observed in the present study are attributed to the prior exercise bout that stimulates amino acid incorporation by skeletal muscle tissue.[5,38,39] Dietary-protein-derived amino acid incorporation into skeletal muscle essentially increased linearly over the entire postprandial following the ingestion of 100 g protein (13 g or 13% of the ingested protein). These data demonstrate that skeletal muscle tissue has a much greater capacity to incorporate exogenous amino acids than previously suggested.

While muscle tissue is known for its plasticity in response to anabolic stimuli, it has a relatively low turnover rate when compared to other tissues.[13,40] Plasma proteins have a very high turnover rate and are believed to have a saturable protein synthetic response to protein ingestion.[18] Therefore, we also assessed the amount of ingested protein incorporated into plasma protein and plasma protein synthesis rates. Dietary-protein-derived amino acid incorporation into plasma protein was estimated to average 2 and 4 g over the 12-h period following ingestion of 25 and 100 g protein, respectively (representing 6% and 4% of the ingested protein, respectively) (Figures 6B and 6D). These data demonstrate that incorporation into plasma protein represents a minor metabolic fate of ingested protein when compared to skeletal muscle tissue. However, these values are relatively high when considering the small plasma protein pool size. This is explained by an up to 8-fold higher plasma protein synthesis rate when compared to muscle tissue. Plasma protein synthesis rates showed similar dose-response patterns (100PRO > 25PRO > 0PRO) over both the initial 4-h period as well as the full 12-h postprandial period (Figure 6E). Plasma protein synthesis rates were approximately 14% and 20% higher following ingestion of 100 compared to 25 g protein over the initial 4-h and full 12-h periods, respectively. In line with muscle and whole-body protein, these data demonstrate that larger amounts of protein increase the magnitude and duration of the plasma protein synthetic response.

Previous work suggests that endogenous-protein-derived amino acids are the main precursors for muscle protein synthesis based on heterogeneity in precursor pool enrichments.[41] Our current observations are in line with and extend these observations

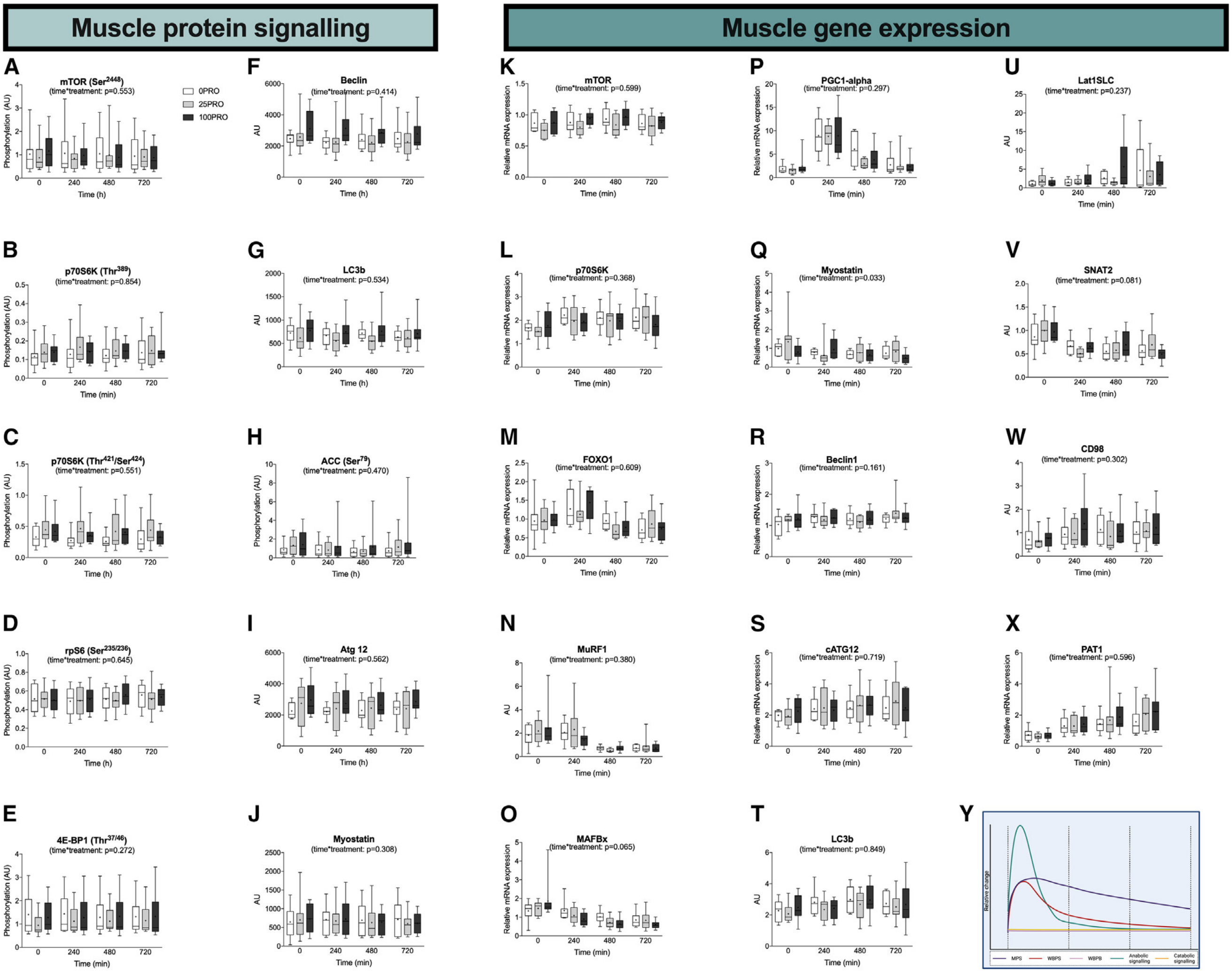

Figure 5. Dissociation between feeding-induced muscle anabolic signaling and protein translation

(A–J) Skeletal muscle phosphorylation status (ratio of phosphorylated to total protein) of mTOR (Ser^{2448}) (A); p70S6K (Thr^{389}) (B); p70S6K (Thr^{421}/Ser^{424}) (C); rpS6 (Ser^{235}/Ser^{236}) (D); 4E-BP1 (Thr^{37}/Thr^{46}) (E); and ACC (Ser^{79}) (H) and protein content of Beclin (F), LC3b (G), Atg 12, (I), and myostatin (J) were all measured by the western blot technique.

(K–X) Skeletal muscle relative mRNA expression (relative to 18S housekeeping gene) of mTOR (K), p70S6K (L), FOXO1 (M), MurF1 (N), MAFBx (O), PGC1-alpha (P), myostatin (Q), Beclin1 (R), cATG12 (S), LC3b (T), LAT1SLC (U), SNAT2 (V), CD98 (W), and PAT1 (X) were all measured by real-time qPCR quantification.

(Y) Conceptual framework of the time course of the muscle protein synthetic, whole-body protein synthetic, whole-body protein breakdown, muscle anabolic signaling, and muscle catabolic signaling response to protein ingestion. Real-time qPCR data are n = 8 (0PRO), 9 (25PRO), and 9 (100PRO) biological replicates. Data in box and whiskers include the median (line), mean (cross), interquartile range (box), and minimum and maximum range (tails).

by assessing the incorporation of exogenous- and endogenous-protein-derived amino acids into muscle tissue protein. We observed that exogenous-protein-derived amino acids contributed 9% and 27% to total amino acid incorporation following the ingestion of a meal-like amount of protein (25PRO) and the large amount of protein (100PRO), respectively (Figure 6H). The higher myofibrillar protein synthesis rates following ingestion of 100 compared to 25 g protein was almost entirely driven by the incorporation of exogenous-protein-derived amino acids. In line, the postprandial increase in amino acid release into the circulation and plasma amino acid uptake into peripheral tissues was almost entirely driven by exogenous-protein-derived amino acids (Figures 6F and 6G). Despite the dose-dependent increase in exogenous-protein-derived amino acid incorporation, endogenous amino acids provided a greater contribution to total amino acid incorporation in the postprandial state (91% and 73% in 25PRO and 100PRO, respectively). Collectively, these data show that the ingestion of a large amount of protein requires a prolonged time period to allow complete protein digestion, amino acid absorption, exogenous-protein-derived amino acid release into the circulation, and subsequent amino acid incorporation into tissue protein pools. Therefore, the duration of the postprandial state is dependent on the amount of ingested protein, with the capacity to exceed 12 h.

CellPress
OPEN ACCESS

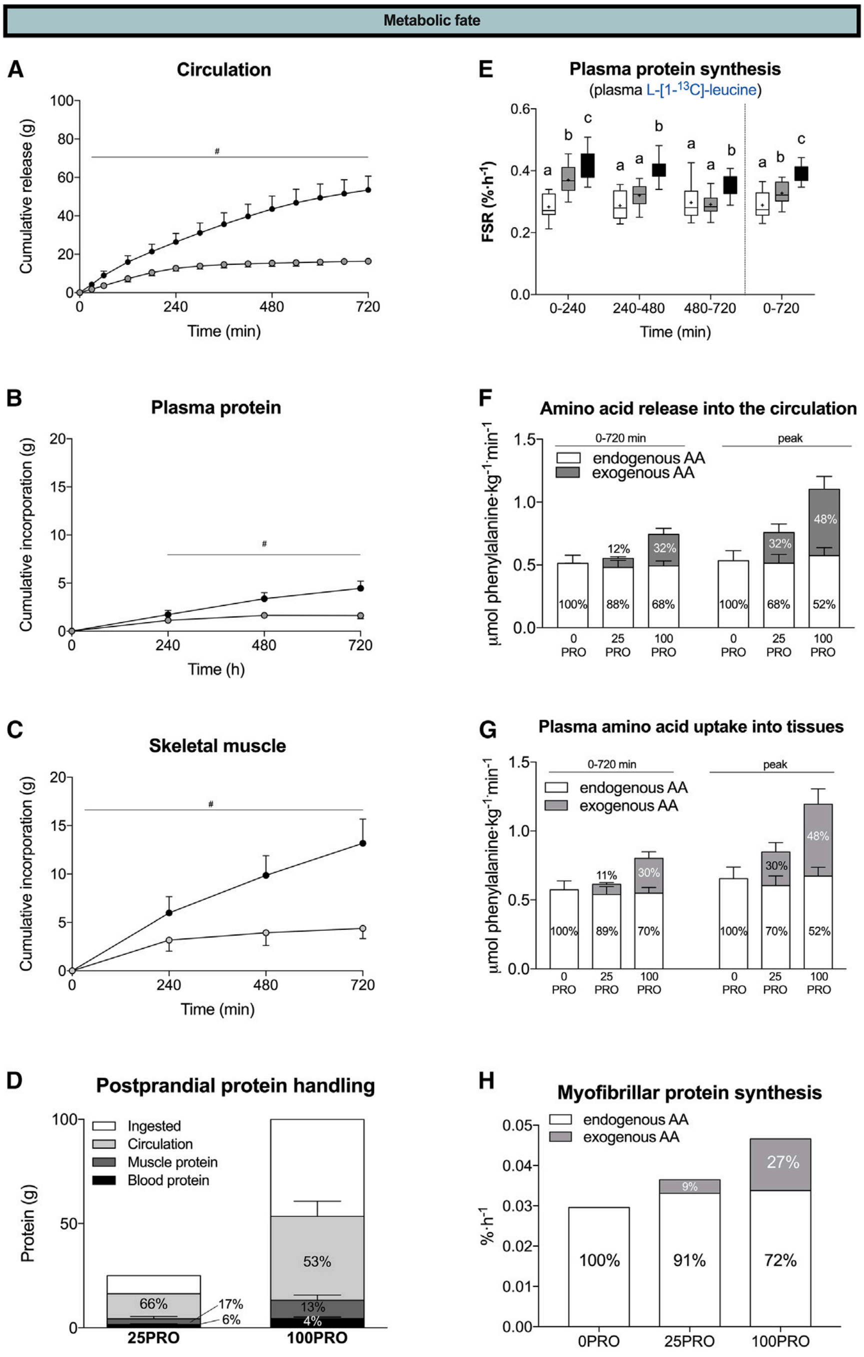

Figure 6. Isotope tracing reveals the dose- and time-dependent metabolic fate of ingested-protein-derived amino acids

(A–C) Cumulative amount of ingested protein that is released into the circulation (A), incorporated into plasma protein (B), and incorporated into skeletal muscle (C).

(D) Metabolic fate of ingested protein expressed in absolute and relative amounts.

(E) Plasma protein synthesis rates based on L-[1-^{13}C]-leucine and the plasma precursor pool.

(F–H) Contribution of endogenous and exogenous rates to the total rate of appearance (F), rate of disappearance (G), and muscle protein synthesis rate (H).

All values are means ± SD. For all measurements, n = 12 biological replicates.

DISCUSSION

Here, we show that the anabolic response to protein ingestion has no apparent upper limit in magnitude and duration *in vivo* in humans. We demonstrate that protein ingestion results in a dose-dependent increase in dietary-protein-derived amino acid availability and a concomitant increase in muscle and whole-body protein synthesis rates (Figure 7). The postprandial increase in plasma amino acid availability has a negligible impact on whole-body protein breakdown or postprandial amino acid oxidation rates. The postprandial increase in muscle protein synthesis rates following ingestion of a large amount of protein (100 g) was sustained well beyond the transient anabolic and catabolic myocellular signaling response to feeding. These data provide valuable mechanistic insight into the ongoing controversy of the impact of different feeding strategies as a means to optimize muscle tissue anabolism and/or metabolic health.

It is generally believed that the ingestion of ∼20 g protein maximizes postprandial muscle protein synthesis rates at rest and during recovery from exercise in healthy, young adults. However, the clinical evidence for this is based on dose-response studies with a small range of protein intakes (≤45 g) and relative short assessment periods (≤6 h).[18–20] Here, we comprehensively assessed the time course of postprandial protein handling by applying a quadruple isotope tracer feeding-infusion approach *in vivo* in humans. To investigate potential upper limits and/or sustained elevation of postprandial protein metabolism, we provided the proposed optimal amount (25 g) and the largest amount of protein that we consider feasible to consume in a single meal (100 g) and evaluated postprandial protein handling throughout a prolonged 12-h assessment period. We observed higher plasma, muscle, and whole-body protein synthesis rates following the ingestion of 100 g protein when compared to 25 g protein and placebo, respectively. The greater metabolic responses were present during the early postprandial phase (0–4 h) but were even more pronounced during the prolonged postprandial phase (4–12 h). These data support our hypothesis that even very large amounts of dietary protein are effectively

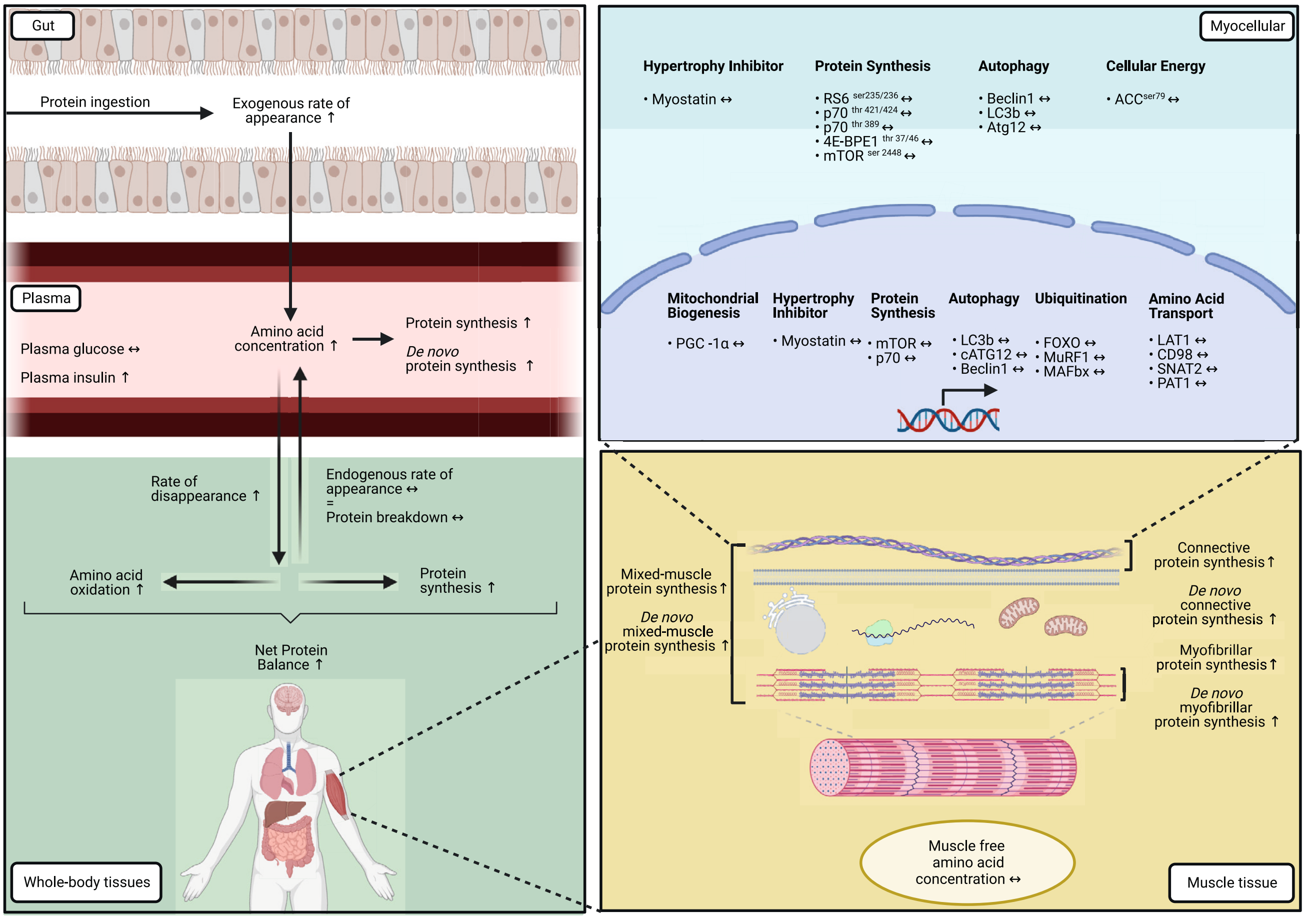

Figure 7. Illustration of the postprandial protein handling

Illustration of study results. Following protein ingestion, exogenous-protein-derived amino acids are released into the circulation, resulting in increased plasma amino acid concentrations. Subsequently, amino acids are taken up by tissues, resulting in an increase in whole-body protein synthesis and net balance, with negligible impact on amino acid oxidation. Protein ingestion does not expand the muscle free amino acid pool but stimulates (*de novo*) muscle protein synthesis rates. Postprandial myocellular protein signaling and gene expression become dissociated from the sustained postprandial increase in muscle protein synthesis rates. The magnitude and duration of the metabolic responses are proportional to the ingested amount of protein.

Arrows are based on the statistical significance of the current study with n = 12 biological replicates for all measurements with the exception of the real-time qPCR data, which were n = 8 or 9.

utilized to support postprandial tissue anabolism but require a more prolonged period for complete protein digestion and amino acid absorption to become available for incorporation into tissues.

It has been proposed that when dietary protein is consumed beyond the rate by which it can be utilized for protein synthesis, the excess amino acids will be directed toward oxidation.[13,32] Our data do not provide any evidence for an upper limit to the whole-body protein synthetic response and, therefore, any disproportional increase in amino acid oxidation following the ingestion of a large amount of protein. In fact, postprandial amino acid oxidation rates were negligible when expressed relative to the increase in whole-body protein synthesis rates (Figure 2J). Therefore, we reassessed amino acid oxidation rates reported in previous dose-response studies by expressing them relative to protein intake.[18,19] This revealed that postprandial amino acid oxidation rates represent a relative minor metabolic fate (<15% of the increment in ingested protein), supporting our findings that the majority of the ingested protein (>85%) is utilized for tissue protein synthesis. Furthermore, these findings seem robust, as they have been observed following ingestion of different types of protein while using different assessment techniques and amino acid tracers to assess amino acid oxidation rates (phenylalanine hydroxylation in the present study and by Witard et al.[19] and breath $^{13}CO_2$ production from leucine by Moore et al.[18]). Collectively, these data refute the notion that amino acid oxidation functions as a sink for excess amino acid provision.

In the present study, milk protein was applied because it is the food item with the largest contribution to daily protein intake in the Western world.[42] Because milk protein consists of 20% rapid digestible whey protein and 80% slowly digestible casein protein, it could be speculated that a more prolonged anabolic response to protein ingestion is unique to slowly digestible

proteins. More rapidly digestible proteins may be disproportionally oxidized and, as such, may result in less amino acids being available for *de novo* protein synthesis.[3] However, previous dose-response studies that administered rapidly digestible protein observed that postprandial amino acid oxidation is actually very limited.[18,19] Moreover, we observed no differences in muscle protein synthesis rates following the ingestion of relatively large doses of whey and casein protein,[43] implying that more rapidly digestible proteins do not result in disproportional (high) amino acid oxidation rates. Collectively, these data indicate that the metabolic responses observed in the present study are unlikely to be restricted to more slowly digestible proteins and are generalizable to other proteins despite different digestion kinetics.

It has been well established that leucine is a key regulator of the mTOR1 pathway in various tissues.[17] We observed that the ingestion of a large amount of protein resulted in an increase in plasma amino acid availability (including leucine). This increase in plasma amino acid availability was not mirrored by an increase in free amino acid concentrations in muscle tissue, as only muscle free branched-chain amino acids concentrations (leucine, isoleucine, and valine) were elevated. Despite the sustained intra- and extracellular increase in leucine availability, the postprandial increase in anabolic myocellular signaling was transient. These data support prior evidence that increased leucine availability plays a critical role in the initial (<4 h) stimulation of postprandial tissue anabolism. Importantly, we extend on this work by showing that prolonged leucine availability does not result in prolonged mTOR activation and that enhanced anabolic signaling is not required to perpetuate the postprandial increase in muscle protein synthesis rate.

We observed that the plasma, muscle, and whole-body protein synthetic responses increased in relation to the magnitude and timeline of the postprandial amino acid response to feeding. This implies that following the activation of anabolic signaling by the postprandial increase in leucine availability, protein synthesis rates are mainly modulated by the availability of amino acids as substrate. In line, we observed that the anabolic responses to feeding corresponded to postprandial plasma amino kinetics (i.e., rate of plasma amino acid appearance and disappearance). Furthermore, we demonstrate for the first time that protein ingestion has a minor impact on endogenous amino acid fluxes, with exogenous amino acid fluxes responding in line with the amount of protein ingested. The relative contribution of exogenous-protein-derived amino acids to overall amino acid release into the circulation, plasma amino acid uptake into tissues, and amino acid incorporation into myofibrillar protein were remarkably similar (32%, 30%, and 27%, respectively, following ingestion of 100 g protein). Collectively, these data demonstrate that protein ingestion results in a coordinated flux of exogenous-protein-derived amino acids toward incorporation in tissue protein that is additive to the endogenous amino acid flux.

Meal frequency has been proposed as an important modulator of tissue and whole-body metabolism. For example, dietary guidelines in both health and disease typically recommend an equal distribution of daily protein requirements over the main meals to support muscle anabolism.[21,22] These recommendations are based entirely on the belief that the muscle protein synthetic response to ingestion of a single bolus of protein has a ceiling and is short lived. The current findings provide evidence to support more flexibility in feeding patterns aimed at enhancing muscle anabolism. Specifically, we show that the ingestion of a single large amount of protein is followed by a prolonged anabolic response, which would obviate the need to consume another protein-rich meal in close temporal proximity. This may explain why time-restricted feeding patterns do not seem to compromise muscle mass maintenance.[44–46] Our data suggest that time-restricted feeding may not result in a postabsorptive state until far beyond the end of the feeding window. This is of relevance since time-restricted feeding is typically applied to avoid prolonged postprandial periods, which are believed to be undesirable for metabolic health.[47,48] Furthermore, it has been speculated that sustained anabolism and mTOR activation inhibit clearance of compromised proteins.[49] However, we observed that the ingestion of a single, large amount of protein (100 g) resulted in prolonged anabolism without compromising whole-body protein breakdown rates, muscle mTOR signaling, or markers of muscle autophagy. Collectively, our data suggest that time-restricted feeding protocols likely overestimate the length of their postabsorptive windows but also that a postabsorptive state is not required to allow protein clearance.

Limitations of the study

The ingestion of 100 g protein resulted in several prolonged metabolic responses that did not return to baseline values at the end of the 12-h assessment period. Therefore, the cumulative metabolic responses are likely even bigger than we were able to observe, and our assessment of the metabolic fate of ingesting 100 g should be considered minimal estimates. A second limitation is that tracer recycling can occur during prolonged amino acid tracer infusion.[50] Therefore, our tracer-derived metabolic rates may be slight underestimations. The degree of underestimation would correlate with the dose-dependent tracer incorporation rates (100PRO > 25PRO > 0PRO). Consequently, the magnitude of difference between treatments would be even greater than observed. The measurement of AA-tRNA, as the preferred precursor pool, is generally not assessed, as the required amount of muscle tissue is not feasible for routine analyses. Therefore, the presented (absolute) muscle protein synthesis rates may be regarded as slight underestimations. However, we demonstrate that the observed muscle protein synthetic response is robust across various fractions, tracers, and precursors pools. We observed a consistent anabolic response at the composite whole-body, muscle, and plasma protein level. However, it should be noted that these protein synthetic responses may not be similar or may even move in the same direction when assessed within different (organ) tissues and between different (sets) of proteins within these compartments.[51] As the present work was acute in nature, it is possible that the metabolic response to protein ingestion is modified over time. For example, it could be speculated that amino acid oxidation may increase in response to frequent, prolonged episodes of hyperaminoacidemia or that large postprandial protein gains are compensated for by upregulation of amino acid oxidation in a subsequent fasted state.[52] Our data provide a mechanistic explanation for why larger but less frequent meals are a

viable alternative to consuming smaller meals more frequently. A final consideration is that we investigated healthy, young men following a bout of whole-body resistance exercise and do not know if our observations can be extrapolated to other populations and/or conditions. It has been well established that the anabolic sensitivity to amino acids is lower in individuals that are more clinically compromised and/or have lower physical activity levels.[53] While there are data that suggest that a blunted anabolic response to feeding in these populations can be compensated for by the ingestion of larger amounts of protein,[54] it is possible that these more clinically compromised individuals may experience a ceiling response in the magnitude and/or duration of the anabolic response to protein ingestion.

STAR★METHODS

Detailed methods are provided in the online version of this paper and include the following:

- KEY RESOURCES TABLE
- RESOURCE AVAILABILITY
  - Lead contact
  - Materials availability
  - Data and code availability
- EXPERIMENTAL MODEL AND STUDY PARTICIPANT DETAILS
  - Subjects
  - Pretesting
  - Diet and physical activity
  - Study design
  - Production of intrinsically labeled milk protein
  - Experimental protocol
  - Exercise protocol
- METHOD DETAILS
  - Plasma and muscle free concentrations
  - Plasma and muscle free enrichments
  - Mixed-muscle tissue analyses
  - Myofibrillar and muscle connective protein analyses
  - Plasma protein analyses
  - Western blotting
  - mRNA analyses
  - Calculations
- QUANTIFICATION AND STATISTICAL ANALYSIS
- ADDITIONAL RESOURCES

SUPPLEMENTAL INFORMATION

Supplemental information can be found online at https://doi.org/10.1016/j.xcrm.2023.101324.

ACKNOWLEDGMENTS

We acknowledge and thank all subjects participating in the study.

AUTHOR CONTRIBUTIONS

J.T., G.A.A.v.L., J.N., J.M.S., and A.M.H. performed animal and/or human experiments. J.S.J.S. and F.K.H. performed muscle biopsies and had the medical responsibility. J.M.S., J.M.X.v.K., J.P.B.G., A.H.Z., and A.P.G. performed the sample analyses. J.T. conceived the hypothesis and performed the calculations and statistical analyses. J.T., A.M.H., and L.J.C.v.L. designed the study. J.T. and L.J.C.v.L. wrote the first draft of manuscript. All authors approved the final version of the manuscript.

DECLARATION OF INTERESTS

G.A.A.v.L. is an employee of FrieslandCampina. The company had no role in funding, data collection and analysis, or preparation of the manuscript. See the following pages for a full overview of career funding for J.T. and L.J.C.v.L.: https://www.maastrichtuniversity.nl/j-trommelen and https://www.maastrichtuniversity.nl/ljc-van-loon.

INCLUSION AND DIVERSITY

We support inclusive, diverse, and equitable conduct of research.

Received: April 26, 2023
Revised: July 3, 2023
Accepted: November 16, 2023
Published: December 19, 2023

REFERENCES

1. Phillips, S.M., Tipton, K.D., Aarsland, A., Wolf, S.E., and Wolfe, R.R. (1997). Mixed muscle protein synthesis and breakdown after resistance exercise in humans. Am. J. Physiol. *273*, E99–E107.
2. Biolo, G., Tipton, K.D., Klein, S., and Wolfe, R.R. (1997). An abundant supply of amino acids enhances the metabolic effect of exercise on muscle protein. Am. J. Physiol. *273*, E122–E129.
3. Trommelen, J., Betz, M.W., and van Loon, L.J.C. (2019). The Muscle Protein Synthetic Response to Meal Ingestion Following Resistance-Type Exercise. Sports Med. *49*, 185–197.
4. Groen, B.B.L., Horstman, A.M., Hamer, H.M., de Haan, M., van Kranenburg, J., Bierau, J., Poeze, M., Wodzig, W.K.W.H., Rasmussen, B.B., and van Loon, L.J.C. (2015). Post-Prandial Protein Handling: You Are What You Just Ate. PLoS One *10*, e0141582.
5. Trommelen, J., Holwerda, A.M., Kouw, I.W.K., Langer, H., Halson, S.L., Rollo, I., Verdijk, L.B., and VAN Loon, L.J.C. (2016). Resistance Exercise Augments Postprandial Overnight Muscle Protein Synthesis Rates. Med. Sci. Sports Exerc. *48*, 2517–2525.
6. Trommelen, J., Holwerda, A.M., Pinckaers, P.J.M., and van Loon, L.J.C. (2021). Comprehensive assessment of post-prandial protein handling by the application of intrinsically labelled protein in vivo in human subjects. Proc. Nutr. Soc. *80*, 221–229.
7. Trommelen, J., Tomé, D., and van Loon, L.J. (2021). Gut amino acid absorption in humans: concepts and relevance for postprandial metabolism. Clin. Nutr. Open Sci. *36*, 43–55.
8. Kashyap, S., Varkey, A., Shivakumar, N., Devi, S., Reddy B H, R., Thomas, T., Preston, T., Sreeman, S., and Kurpad, A.V. (2019). True ileal digestibility of legumes determined by dual-isotope tracer method in Indian adults. Am. J. Clin. Nutr. *110*, 873–882.
9. Weijzen, M.E.G., van Gassel, R.J.J., Kouw, I.W.K., Trommelen, J., Gorissen, S.H.M., van Kranenburg, J., Goessens, J.P.B., van de Poll, M.C.G., Verdijk, L.B., and van Loon, L.J.C. (2022). Ingestion of Free Amino Acids Compared with an Equivalent Amount of Intact Protein Results in More Rapid Amino Acid Absorption and Greater Postprandial Plasma Amino Acid Availability Without Affecting Muscle Protein Synthesis Rates in Young Adults in a Double-Blind Randomized Trial. J. Nutr. *152*, 59–67.
10. Volpi, E., Mittendorfer, B., Wolf, S.E., and Wolfe, R.R. (1999). Oral amino acids stimulate muscle protein anabolism in the elderly despite higher first-pass splanchnic extraction. Am. J. Physiol. *277*, E513–E520.
11. Boirie, Y., Gachon, P., and Beaufrère, B. (1997). Splanchnic and whole-body leucine kinetics in young and elderly men. Am. J. Clin. Nutr. *65*, 489–495.

12. Gorissen, S.H.M., Trommelen, J., Kouw, I.W.K., Holwerda, A.M., Pennings, B., Groen, B.B.L., Wall, B.T., Churchward-Venne, T.A., Horstman, A.M.H., Koopman, R., et al. (2020). Protein Type, Protein Dose, and Age Modulate Dietary Protein Digestion and Phenylalanine Absorption Kinetics and Plasma Phenylalanine Availability in Humans. J. Nutr. *150*, 2041–2050.

13. Trommelen, J., and van Loon, L.J.C. (2021). Assessing the whole-body protein synthetic response to feeding in vivo in human subjects. Proc. Nutr. Soc. *80*, 139–147.

14. Bar-Peled, L., and Sabatini, D.M. (2014). Regulation of mTORC1 by amino acids. Trends Cell Biol. *24*, 400–406.

15. Moro, T., Ebert, S.M., Adams, C.M., and Rasmussen, B.B. (2016). Amino Acid Sensing in Skeletal Muscle. Trends Endocrinol. Metab. *27*, 796–806.

16. Rundqvist, H.C., Esbjörnsson, M., Rooyackers, O., Österlund, T., Moberg, M., Apro, W., Blomstrand, E., and Jansson, E. (2017). Influence of nutrient ingestion on amino acid transporters and protein synthesis in human skeletal muscle after sprint exercise. J. Appl. Physiol. *123*, 1501–1515. 1985.

17. Son, S.M., Park, S.J., Lee, H., Siddiqi, F., Lee, J.E., Menzies, F.M., and Rubinsztein, D.C. (2019). Leucine Signals to mTORC1 via Its Metabolite Acetyl-Coenzyme A. Cell Metab. *29*, 192–201.e7.

18. Moore, D.R., Robinson, M.J., Fry, J.L., Tang, J.E., Glover, E.I., Wilkinson, S.B., Prior, T., Tarnopolsky, M.A., and Phillips, S.M. (2009). Ingested protein dose response of muscle and albumin protein synthesis after resistance exercise in young men. Am. J. Clin. Nutr. *89*, 161–168.

19. Witard, O.C., Jackman, S.R., Breen, L., Smith, K., Selby, A., and Tipton, K.D. (2014). Myofibrillar muscle protein synthesis rates subsequent to a meal in response to increasing doses of whey protein at rest and after resistance exercise. Am. J. Clin. Nutr. *99*, 86–95.

20. Churchward-Venne, T.A., Pinckaers, P.J.M., Smeets, J.S.J., Betz, M.W., Senden, J.M., Goessens, J.P.B., Gijsen, A.P., Rollo, I., Verdijk, L.B., and van Loon, L.J.C. (2020). Dose-response effects of dietary protein on muscle protein synthesis during recovery from endurance exercise in young men: a double-blind randomized trial. Am. J. Clin. Nutr. *112*, 303–317.

21. Phillips, S.M., and Van Loon, L.J.C. (2011). Dietary protein for athletes: from requirements to optimum adaptation. J. Sports Sci. *29* (*Suppl 1*), S29–S38.

22. Murphy, C.H., Oikawa, S.Y., and Phillips, S.M. (2016). Dietary Protein to Maintain Muscle Mass in Aging: A Case for Per-meal Protein Recommendations. J. Frailty Aging *5*, 49–58.

23. McCue, M.D. (2006). Specific dynamic action: a century of investigation. Comp. Biochem. Physiol. Mol. Integr. Physiol. *144*, 381–394.

24. Cox, C.L., and Secor, S.M. (2010). Integrated postprandial responses of the diamondback water snake, Nerodia rhombifer. Physiol. Biochem. Zool. *83*, 618–631.

25. McCue, M.D., Bennett, A.F., and Hicks, J.W. (2005). The effect of meal composition on specific dynamic action in Burmese pythons (Python molurus). Physiol. Biochem. Zool. *78*, 182–192.

26. McCue, M.D., Guzman, R.M., and Passement, C.A. (2015). Digesting pythons quickly oxidize the proteins in their meals and save the lipids for later. J. Exp. Biol. *218*, 2089–2096.

27. Wang, T., and Rindom, E. (2021). The physiological response to digestion in snakes: a feast for the integrative physiologist. Comp. Biochem. Physiol. Mol. Integr. Physiol. *254*, 110891.

28. van Loon, L.J.C., Boirie, Y., Gijsen, A.P., Fauquant, J., de Roos, A.L., Kies, A.K., Lemosquet, S., Saris, W.H.M., and Koopman, R. (2009). The production of intrinsically labeled milk protein provides a functional tool for human nutrition research. J. Dairy Sci. *92*, 4812–4822.

29. Church, D.D., Hirsch, K.R., Park, S., Kim, I.Y., Gwin, J.A., Pasiakos, S.M., Wolfe, R.R., and Ferrando, A.A. (2020). Essential Amino Acids and Protein Synthesis: Insights into Maximizing the Muscle and Whole-Body Response to Feeding. Nutrients *12*, 3717.

30. Bohé, J., Low, A., Wolfe, R.R., and Rennie, M.J. (2003). Human muscle protein synthesis is modulated by extracellular, not intramuscular amino acid availability: a dose-response study. J. Physiol. *552*, 315–324.

31. Bergström, J., Fürst, P., and Hultman, E. (1985). Free amino acids in muscle tissue and plasma during exercise in man. Clin. Physiol. *5*, 155–160.

32. Elango, R., Ball, R.O., and Pencharz, P.B. (2008). Indicator amino acid oxidation: concept and application. J. Nutr. *138*, 243–246.

33. Tipton, K.D., Ferrando, A.A., Phillips, S.M., Doyle, D., Jr., and Wolfe, R.R. (1999). Postexercise net protein synthesis in human muscle from orally administered amino acids. Am. J. Physiol. *276*, E628–E634.

34. Holwerda, A.M., Trommelen, J., Kouw, I.W.K., Senden, J.M., Goessens, J.P.B., van Kranenburg, J., Gijsen, A.P., Verdijk, L.B., and van Loon, L.J.C. (2021). Exercise Plus Presleep Protein Ingestion Increases Overnight Muscle Connective Tissue Protein Synthesis Rates in Healthy Older Men. Int. J. Sport Nutr. Exerc. Metab. *31*, 217–226.

35. Trommelen, J., Holwerda, A.M., Senden, J.M., Goessens, J.P.B., VAN Kranenburg, J., Gijsen, A.P., Verdijk, L.B., and VAN Loon, L.J.C. (2020). Casein Ingestion Does Not Increase Muscle Connective Tissue Protein Synthesis Rates. Med. Sci. Sports Exerc. *52*, 1983–1991.

36. Dideriksen, K.J., Reitelseder, S., Petersen, S.G., Hjort, M., Helmark, I.C., Kjaer, M., and Holm, L. (2011). Stimulation of muscle protein synthesis by whey and caseinate ingestion after resistance exercise in elderly individuals. Scand. J. Med. Sci. Sports *21*, e372–e383.

37. Camera, D.M., West, D.W.D., Phillips, S.M., Rerecich, T., Stellingwerff, T., Hawley, J.A., and Coffey, V.G. (2015). Protein ingestion increases myofibrillar protein synthesis after concurrent exercise. Med. Sci. Sports Exerc. *47*, 82–91.

38. Holwerda, A.M., Kouw, I.W., Trommelen, J., Halson, S.L., Wodzig, W.K., Verdijk, L.B., and van Loon, L.J. (2016). Physical Activity Performed in the Evening Increases the Overnight Muscle Protein Synthetic Response to Presleep Protein Ingestion in Older Men. J. Nutr. *146*, 1307–1314.

39. Pennings, B., Koopman, R., Beelen, M., Senden, J.M.G., Saris, W.H.M., and van Loon, L.J.C. (2011). Exercising before protein intake allows for greater use of dietary protein-derived amino acids for de novo muscle protein synthesis in both young and elderly men. Am. J. Clin. Nutr. *93*, 322–331.

40. van Dijk, D.P.J., Horstman, A.M.H., Smeets, J.S.J., den Dulk, M., Grabsch, H.I., Dejong, C.H.C., Rensen, S.S., Olde Damink, S.W.M., and van Loon, L.J.C. (2019). Tumour-specific and organ-specific protein synthesis rates in patients with pancreatic cancer. J. Cachexia Sarcopenia Muscle *10*, 549–556.

41. Ljungqvist, O.H., Persson, M., Ford, G.C., and Nair, K.S. (1997). Functional heterogeneity of leucine pools in human skeletal muscle. Am. J. Physiol. *273*, E564–E570.

42. Gorissen, S.H.M., and Witard, O.C. (2018). Characterising the muscle anabolic potential of dairy, meat and plant-based protein sources in older adults. Proc. Nutr. Soc. *77*, 20–31.

43. Trommelen, J., van Lieshout, G.A.A., Pabla, P., Nyakayiru, J., Hendriks, F.K., Senden, J.M., Goessens, J.P.B., van Kranenburg, J.M.X., Gijsen, A.P., Verdijk, L.B., et al. (2023). Pre-sleep Protein Ingestion Increases Mitochondrial Protein Synthesis Rates During Overnight Recovery from Endurance Exercise: A Randomized Controlled Trial. Sports Med. *53*, 1445–1455.

44. Trepanowski, J.F., Kroeger, C.M., Barnosky, A., Klempel, M.C., Bhutani, S., Hoddy, K.K., Gabel, K., Freels, S., Rigdon, J., Rood, J., et al. (2017). Effect of Alternate-Day Fasting on Weight Loss, Weight Maintenance, and Cardioprotection Among Metabolically Healthy Obese Adults: A Randomized Clinical Trial. JAMA Intern. Med. *177*, 930–938.

45. Gu, L., Fu, R., Hong, J., Ni, H., Yu, K., and Lou, H. (2022). Effects of Intermittent Fasting in Human Compared to a Non-intervention Diet and Caloric Restriction: A Meta-Analysis of Randomized Controlled Trials. Front. Nutr. *9*, 871682.

46. Tinsley, G.M., Moore, M.L., Graybeal, A.J., Paoli, A., Kim, Y., Gonzales, J.U., Harry, J.R., VanDusseldorp, T.A., Kennedy, D.N., and Cruz, M.R. (2019). Time-restricted feeding plus resistance training in active females: a randomized trial. Am. J. Clin. Nutr. *110*, 628–640.

47. Martinez-Lopez, N., Tarabra, E., Toledo, M., Garcia-Macia, M., Sahu, S., Coletto, L., Batista-Gonzalez, A., Barzilai, N., Pessin, J.E., Schwartz, G.J., et al. (2017). System-wide Benefits of Intermeal Fasting by Autophagy. Cell Metab. *26*, 856–871.e5.

48. Sutton, E.F., Beyl, R., Early, K.S., Cefalu, W.T., Ravussin, E., and Peterson, C.M. (2018). Early Time-Restricted Feeding Improves Insulin Sensitivity, Blood Pressure, and Oxidative Stress Even without Weight Loss in Men with Prediabetes. Cell Metab. *27*, 1212–1221.e3.

49. Saxton, R.A., and Sabatini, D.M. (2017). mTOR Signaling in Growth, Metabolism, and Disease. Cell *168*, 960–976.

50. Schwenk, W.F., Tsalikian, E., Beaufrere, B., and Haymond, M.W. (1985). Recycling of an amino acid label with prolonged isotope infusion: implications for kinetic studies. Am. J. Physiol. *248*, E482–E487.

51. De Feo, P., Volpi, E., Lucidi, P., Cruciani, G., Reboldi, G., Siepi, D., Mannarino, E., Santeusanio, F., Brunetti, P., and Bolli, G.B. (1993). Physiological increments in plasma insulin concentrations have selective and different effects on synthesis of hepatic proteins in normal humans. Diabetes *42*, 995–1002.

52. Walrand, S., Short, K.R., Bigelow, M.L., Sweatt, A.J., Hutson, S.M., and Nair, K.S. (2008). Functional impact of high protein intake on healthy elderly people. Am. J. Physiol. Endocrinol. Metab. *295*, E921–E928.

53. Paulussen, K.J.M., McKenna, C.F., Beals, J.W., Wilund, K.R., Salvador, A.F., and Burd, N.A. (2021). Anabolic Resistance of Muscle Protein Turnover Comes in Various Shapes and Sizes. Front. Nutr. *8*, 615849.

54. Wall, B.T., Gorissen, S.H., Pennings, B., Koopman, R., Groen, B.B.L., Verdijk, L.B., and van Loon, L.J.C. (2015). Aging Is Accompanied by a Blunted Muscle Protein Synthetic Response to Protein Ingestion. PLoS One *10*, e0140903.

55. Mayhew, J.L., Prinster, J.L., Ware, J.S., Zimmer, D.L., Arabas, J.R., and Bemben, M.G. (1995). Muscular endurance repetitions to predict bench press strength in men of different training levels. J. Sports Med. Phys. Fitness *35*, 108–113.

56. van Loon, L.J.C., Boirie, Y., Gijsen, A.P., Fauquant, J., de Roos, A.L., Kies, A.K., Lemosquet, S., Saris, W.H.M., and Koopman, R. (2009). The production of intrinsically labeled milk protein provides a functional tool for human nutrition research. J. Dairy Sci. *92*, 4812–4822.

57. Boirie, Y., Gachon, P., Corny, S., Fauquant, J., Maubois, J.L., and Beaufrère, B. (1996). Acute postprandial changes in leucine metabolism as assessed with an intrinsically labeled milk protein. Am. J. Physiol. *271*, E1083–E1091.

CellPress
OPEN ACCESS

STAR★METHODS

KEY RESOURCES TABLE

REAGENT or RESOURCE	SOURCE	IDENTIFIER
Antibodies		
Phospho mTOR (Ser 2448)	Cell Signaling	Cat#2971; AB_330970
mTor	Cell Signaling	Cat#2972; AB_330978
Phospho p70 S6 (Thr 389)	Cell Signaling	Cat#9205; AB_330944
Phospho p70 S6 (Thr 421/424)	Cell Signaling	Cat#9204; AB_2265913
P70 S6	Cell Signaling	Cat#9202; AB_331676
Phospho rS6 (Ser 235/236)	Cell Signaling	Cat#4856; AB_2181037
Phospho rS6 (Ser240/244)	Cell Signaling	Cat#2215; AB_331682
rS6	Cell Signaling	Cat#2217; AB_331355
Phospho ACC (Ser 79)	Cell Signaling	Cat#3661; AB_330337
ACC	Cell Signaling	Cat#3662; AB_2219400
Phospho 4E-BP1 (Thr 37/64)	Cell Signaling	Cat#9459; AB_330985
4E-BP1	Cell Signaling	Cat#9452; AB_331692
Myostatin	Santa Cruz	Sc134345; AB_2148785
Beclin	Cell Signaling	Cat#3738; AB_490837
LC3b	Cell Signaling	Cat#2775; AB_915950
Atg12	Cell Signaling	Cat#4180; AB_1903898
Chemicals, peptides and recombinant proteins		
L-phenylalanine (1-^{13} C)	Cambridge Isotopes	CLM-762-MPT-PK
L-leucine (1-^{13} C)	Cambridge Isotopes	CLM-468-MPT-PK
L-phenylalanine (ring-D_5)	Cambridge Isotopes	DLM-1258-MPT-PK
L-Tyrosine (ring-3,5-D_2)	Cambridge Isotopes	DLM-449-MPT-PK
[U-$^{13}C_6$]-leucine	Cambridge Isotopes	CLM-2262
[U-$^{13}C_9{}^{15}N$]-phenylalanine	Cambridge Isotopes	CNLM-575
[U-$^{13}C_9{}^{15}N$]-tyrosine	Cambridge Isotopes	CNLM-439
AG 50W-X8 resin, mesh size: 100–200 μm, ionic form: hydrogen	Bio-Rad Laboratories	142–1441
MTBSTFA	Merc	8.14219
Metabolomics Amino Acid Standard	Cambridge Isotopes	MSK-A2-S
Amino Acid Standards Basics	Sigma-Aldrich	A6282
Amino Acid Standards Neutrals + Basics	Sigma-Aldrich	A6407
Accq-Taq ultra derivatization	Waters	186003836
FOXO1 taqman probe	Fisher Emergo	Hs01054576_m1
MuRF1 taqman probe	Fisher Emergo	Hs00261590_m1
MAFbx taqman probe	Fisher Emergo	Hs01041408_m1
mTOR taqman probe	Fisher Emergo	Hs00234508_m1
p70S6K taqman probe	Fisher Emergo	Hs00177357_m1
cATG12 taqman probe	Fisher Emergo	Hs01047860_g1
LC3b taqman probe	Fisher Emergo	Hs00797944_s1
Lat1SLC taqman probe	Fisher Emergo	Hs00185826_m1
PAT1 taqman probe	Fisher Emergo	Hs01092773_m1
SNAT2 taqman probe	Fisher Emergo	Hs01089954_m1
CD98 taqman probe	Fisher Emergo	Hs00374243_m1
Beclin1 taqman probe	Fisher Emergo	Hs00186838_m1
Myostatin taqman probe	Fisher Emergo	Hs00193363_m1
PGC1-alpha taqman probe	Fisher Emergo	Hs01016719_m1

(*Continued on next page*)

Continued		
REAGENT or RESOURCE	SOURCE	IDENTIFIER
18S taqman probe	Fisher Emergo	Hs03003631_g1
Critical commercial assays		
Glucose HK CP	ABX Pentra	A11A01667
Insulin Elisa kit	Meso Scale Discovery	K151BZC-3
Software		
Image Studio 5.2	LI-COR biosciences	5.2
Prism	GraphPad Software	v7.0a
SPSS	IBM	27.0.1.0
BioRender	BioRender	https://www.biorender.com/

RESOURCE AVAILABILITY

Lead contact

Further information and requests for resources and reagents as well as datasets and protocols should be directed to and will be fulfilled by the Lead Contact, Luc J.C. van Loon (l.vanloon@maastrichtuniversity.nl).

Materials availability

This study did not generate new unique reagents.

Data and code availability

- All data reported in this paper will be shared by the lead contact upon request.
- This study does not report original code.
- Any additional information required to reanalyze the data reported in this work paper is available from the lead contact upon request.

EXPERIMENTAL MODEL AND STUDY PARTICIPANT DETAILS

Subjects

A total of 36 healthy, recreationally active young men were selected to participate in this parallel group, randomized double blind placebo-controlled trial. Inclusion criteria were: male, aged between 18 and 40 years, healthy, and body mass index between 18.5 and 30 kg m^{-2}. Exclusion criteria were: smoking, sports/exercise <1 or >3 sessions per week, lactose intolerant, a history of neuromuscular problems, use of anticoagulation medication, and individuals taking any medication known to affect protein metabolism. Subject characteristics are presented in Table S1.

Pretesting

Participants underwent an initial screening session to assess height, weight, and body composition. Body composition was assessed by bioelectric impedance (Star Test protocol, BioScan 920-2, Maltron International, Essex, UK). Participants were deemed healthy based on their responses to a medical questionnaire and screening results. The subjects were then familiarized with the resistance-type exercise protocol and the exercise equipment. All exercises during pretesting and experimental trials were supervised by trained personnel. Subjects started by performing a 5-min cycling warm-up at 100 W before completing an estimation of their 1-repetition maximum (1-RM) on the leg press, leg extension, chest press, and lat pulldown exercises using the multiple repetitions testing procedure.[55] For each exercise, subjects performed 10 submaximal repetitions to become familiarized with the equipment and to have lifting technique critiqued and properly adjusted. Sets were then performed at progressively increasing loads until failure to perform a valid estimation within 3–6 repetitions of the set. A repetition was valid if the subject was able to complete the entire lift in a controlled manner without assistance. A 2-min resting period between subsequent attempts was allowed. The pretesting and experimental trials were separated by at least 7 days.

Diet and physical activity

All subjects were instructed to refrain from exhaustive physical labor and exercise and to keep their diet as constant as possible 2 days before the experimental day. Food intake and physical activity questionnaires were collected for 2 days before the experiment. The evening before the trial, all participants were instructed to consume a prepackaged standardized meal containing 54 kJ kg^{-1} with 60% energy as carbohydrate, 25% energy as fat, and 15% energy as fat. Participants were instructed to finish it before 22:00 h, after which they remained fasted.

Study design

Participants were randomly assigned to ingest a beverage containing 800 mL water with either 0 g, 25 g, or 100 g intrinsically L-[1-^{13}C]-phenylalanine and L-[1-^{13}C]-leucine labeled milk protein (described below). Randomization was performed using a computerized list randomizer (http://www.randomization.com/) and participants were sequentially allocated to a treatment according to the randomized list. An independent person was responsible for random assignment (n = 12 per group) and preparation of the study treatment beverages, which were sequentially numbered according to subject number. The beverages were prepared in a nontransparent plastic container and provided in a double-blind fashion.

Production of intrinsically labeled milk protein

A lactating Holstein cow was selected for the production of intrinsically L-[1-^{13}C]-phenylalanine and L-[1-^{13}C]-leucine labeled milk protein. A constant intravenous infusion of L-[1-^{13}C]-phenylalanine (455 μmol/min) and L-[1-^{13}C]-leucine (200 μmol/min) was applied for 96 h. Milk was collected during and following the infusion period to produce higher and lower labeled milk, respectively. Subsequently, the milk was processed into milk protein concentrate.[56] The protein met all chemical and bacteriological specifications for human consumption.

Experimental protocol

At ~07:45, participants arrived at the laboratory in the overnight postabsorptive state. A catheter was inserted into an antecubital vein for stable isotope amino acid infusion, then a second catheter was subsequently inserted into a dorsal hand vein on the contralateral arm for arterialized venous blood sampling. To obtain arterialized blood samples, the hand was placed in a hot box (60°C) for 10 min before blood sample collection. After taking a baseline blood sample (t = −150 min), the plasma phenylalanine, leucine, and tyrosine pools were primed with a single intravenous dose (priming dose) of L-[ring-$^{2}H_5$]-phenylalanine (3.60 μmol/kg), L-[ring-3,5-$^{2}H_2$]-tyrosine (1.10 μmol/kg), and L-[1-^{13}C]-leucine (7.19 μmol/kg). After priming, a continuous intravenous infusion of L-[ring-$^{2}H_5$]-phenylalanine (0.060 μmol • kg^{-1} • min^{-1}), L-[ring-3,5-$^{2}H_2$]-tyrosine (0.018 μmol • kg^{-1} • min^{-1}), and L-[1-^{13}C]-leucine (0.12 μmol • kg^{-1} • min^{-1}) was initiated and maintained using a calibrated IVAC 598 pump. After resting in a supine position for 90 min, a second arterialized blood sample was drawn (t = −60 min). Subsequently, participants initiated the resistance-type exercise intervention (described below). Immediately after the exercise intervention (t = 0 min), an arterialized blood sample was obtained, and a muscle biopsy sample was collected from the vastus lateralis muscle of a randomly selected leg. Subsequently, participants received their experimental drink. Arterialized blood samples were then collected at t = 30, 60, 120, 180, 240, 300, 360, 420, 480, 540, 600, 660, and 720 min in the postprandial period. Second, third and fourth muscle biopsy samples were collected at t = 240, t = 480, and *t* = 720 min. Blood samples were collected into EDTA-containing tubes and centrifuged at 1000g for 15 min at 4°C. Aliquots of plasma were frozen in liquid nitrogen and stored at −80°C. Biopsy samples were collected using a 5-mm Bergström needle custom-adapted for manual suction. Samples were obtained from separate incisions from the middle region of the vastus lateralis, ~15 cm above the patella and ~3 cm below entry through the fascia, under 1% xylocaine local anesthesia with adrenaline (1:100,000). Muscle samples were freed from any visible non-muscle material, immediately frozen in liquid nitrogen, and stored at −80°C until further processing. For a schematic representation of the primed continuous infusion protocol, see Figure 1A.

Exercise protocol

The exercise protocol consisted of a 60-min whole-body resistance-type exercise bout. Subjects performed a 5 min cycling warm up at 100 W with a cadence of 60–80 rpm. Thereafter, subjects performed four sets of 10 repetitions for the each of the following exercises: leg press machine, the leg extension, the lat pulldown, and the chest press (Technogym BV, Rotterdam, Netherlands). The first of each exercise was performed at 65% of the subjects 1-RM for 10 reps. Sets 2–4 were performed at 80% of 1-RM until volitional fatigue under strong verbal encouragement. There were 2-min rest intervals between sets.

METHOD DETAILS

Plasma and muscle free concentrations

Plasma glucose and insulin concentrations were analyzed using commercially available kits (GLUC3, Roche, ref. 05168791 190, and ref. no. K151BZC-3, MSD Insulin Elisa kit, Meso Scale Discovery, Inc. Rockville, Maryland, respectively). Plasma and muscle free amino acid concentrations were measured using ultra-performance liquid chromatograph-mass spectrometry (UPLC-MS; ACQUITY UPLC H-Class with QDa; Waters, Saint-Quentin, France). For plasma, 50 μL was deproteinized using 100 μL of 10% SSA with 50 μM of MSK-A2 internal standard (Cambridge Isotope Laboratories, Massachusetts, USA). For muscle tissue, at least 5 mg of freeze-dried tissue was hydrolyzed in 3 mL of 6 M HCl for 12 h at 110°C and dried under a continuous N2-stream. 5 mL of 0.1 M HCl was used to reconstitute the hydrolysates after which 50 μL of each protein hydrolysate was deproteinized using 100 μL of 10% SSA with 50 μM of MSK-A2 internal standard (Cambridge Isotope Laboratories, Massachusetts, USA). Subsequently, procedures were identical for plasma and muscle tissue. 50 μL of ultra-pure demineralized water was added and samples were centrifuged. After centrifugation, 10 μL of supernatant was added to 70 μL of Borate reaction buffer (Waters, Saint-Quentin, France). In addition, 20 μL of AccQ-Tag derivatizing reagent solution (Waters, Saint-Quentin, France) was added after which the solution was heated for 10 min at 55°C. Of this 100 μL derivative 1 μL was injected and measured using UPLC-MS.

Plasma and muscle free enrichments

Plasma and muscle free amino acid enrichments were determined by GC-MS (Agilent 7890A GC/5975C; MSD, Little Falls, DE). Specifically, internal standards of [U-$^{13}C_6$]-leucine, [U-$^{13}C_9{}^{15}N$]-phenylalanine, and [U-$^{13}C_9{}^{15}N$]-tyrosine were added to the samples. The samples were deproteinized on ice with dry 5-sulfosalicylic acid. Free amino acids were purified using cation exchange chromatography (AG 50W-X8 resin, mesh size: 100–200 μm, ionic form: hydrogen; Bio-Rad Laboratories, Hercules, CA). The free amino acids were converted to their *tert*-butyl dimethylsilyl (tBDMS) derivatives with MTBSTFA before analysis by GC-MS. The amino acid concentrations were determined using electron impact ionization by monitoring ions at mass/charge (m/z) 302 and 308 for unlabeled and [U-$^{13}C_6$]-leucine, 336 and 346 for unlabeled and [U-$^{13}C_9{}^{15}N$]-phenylalanine, and 466 and 476 for unlabeled and [U-$^{13}C_9{}^{15}N$]-tyrosine respectively. The plasma and muscle free leucine, phenylalanine, and tyrosine ^{13}C and ^{2}H enrichments were determined using selective ion monitoring at m/z 302 and 303 for unlabeled and labeled L-[1-^{13}C]-leucine, respectively; m/z 336, 337, and 341 for unlabeled and labeled L-[1-^{13}C]-phenylalanine and L-[*ring*-$^{2}H_5$]-phenylalanine, respectively; m/z 466, 467, 468, and 470 for unlabeled and labeled L-[1-^{13}C]-tyrosine, L-[*ring*-3,5-$^{2}H_2$]-tyrosine and L-[*ring*-$^{2}H_4$]-tyrosine, respectively. Standard regression curves were applied from a series of known standard enrichment values against the measured values to assess the linearity of the mass spectrometer and to account for any isotope fractionation.

Mixed-muscle tissue analyses

Mixed-muscle protein was extracted from 50 mg wet muscle tissue that was freeze-dried. Collagen, blood, and other non-muscle fiber material were removed from the muscle fibers under a light microscope. The isolated muscle fiber mass (approximately 8 mg) was weighted and 35 volumes (7 times dry weight of isolated muscle fibers wet:dry ratio) of ice-cold 2% PCA were added. Thereafter, the tissue was sonicated and centrifuged. The protein pellet was washed with 3 additional 1.5 mL washes of 2% PCA, hydrolyzed with 6M HCl at 110°C for 15–18 h, and then dried under a nitrogen steam while heated to 110°C. Next, 50% acetic acid solution was added, and the hydrolyzed protein was passed over a cation exchange resin (Dowex AG 50W-X8, 100–200 mesh hydrogen form: Bio-Rad, Hercules, CA) using 2M NH_4OH. The eluate was dried, and the purified amino acids were derivatized to their N(O,S)-ethoxycarbonyl ethyl esters. For the measurement of L-[1-^{13}C]-leucine and L-[1-^{13}C]-phenylalanine, hydrolyzed mixed-muscle protein samples were processed via the same procedures as in the myofibrillar and muscle connective protein analyses described below.

Myofibrillar and muscle connective protein analyses

Muscle connective and myofibrillar protein-enriched fractions were isolated from 50 to 90 mg of wet muscle tissue by hand homogenizing on ice using a pestle in a standard (SET buffer: sucrose, EDTA and tris) extraction buffer (10 μL mg^{-1}). The samples were spun for 15 min at 700*g* and 4°C. The supernatant was transferred to a separate tube for Western blot analysis. The pellet was washed with 400 μL of extraction buffer before vortexing and centrifugation at 700*g* and 4°C for 10 min. The supernatant was removed, and the pellet was washed with 500 μL ddH2O before vortexing and centrifugation at 700*g* and 4°C for 10 min. The supernatant was removed, and 1 mL of homogenization buffer was added and the material was suspended by vortexing before transferring into microtubes containing 1.4 mm ceramic beads and Lysing Matrix D (MP Biomedicals, Irvine, CA). The microtubes were vigorously shaken four times for 45 s at 5.5 m s^{-1} (FastPrep-24 5G, MP Biomedicals) to mechanically lyse the protein network. Samples were then left to rest at 4°C for 3 h before centrifugation at 700*g* and 4°C for 20 min, discarding the supernatant and adding 1 mL of homogenization buffer. The microtubes were shaken for 40 s at 5.5 m s^{-1} before centrifugation at 700*g* and 4°C for 20 min. The supernatant was discarded, and 1 mL of KCl buffer was added to precipitate connective proteins overnight at 4°C. The next morning, samples were vortexed and centrifuged at 1600*g* for 20 min at 4°C. For the myofibrillar isolation the supernatant was transferred to a separate tube. Then, 3.4 mL EtOH 100% were added, samples were vortexed, left for 2 h at 4°C and then centrifuged at 1600g, for 20 min at 4°C. Supernatant was discarded and EtOH 70% was added to the pellet, vortexed and centrifuge again at 1600g, for 20 min at 4°C. Supernatant was again discarded and the remaining pellet was suspended in 2 mL of 6 M HCl in glass screw-cap tubes and left to hydrolyze overnight at 110°C. For the connective isolation, the pellet, containing both immature and mature connective proteins, was mixed with 1 mL KCl buffer and left for 2 h at 4°C. The samples were vortexed and centrifuged at 1600g for 20 min at 4°C, and the supernatant was discarded. To the pellet, 1 mL ddH2O were added, vortexed, left for 2 h at 4°C and then centrifuged at 1600*g*, for 20 min at 4°C. The supernatant was removed and the remaining pellet was suspended in 1 mL of 6 M HCl in glass screw-cap tubes and left to hydrolyze overnight at 110°C. The hydrolyzed protein was then dried under a nitrogen stream when being heated to 110°C.

Following hydrolyzation, the free amino acids were dissolved in 25% acetic acid solution, passed over cation exchange AG 50W-X8 resin columns (mesh size: 100–200, ionic form: hydrogen; Bio-Rad Laboratories, Hercules, CA), washed 5 times with water and finally eluted with 2 M NH4OH. For measurement of L-[1-^{13}C]-leucine and L-[1-^{13}C]-phenylalanine, derivatized samples were measured using a GC-C-IRMS (MAT 253; Thermo Fisher Scientific, Bremen, Germany) equipped with DB5MS 30m column (No. 122–5532; Agilent) and GC-Isolink monitoring of ion masses 44, 45, and 46. For measurement of L-[ring-$^{2}H_5$]-phenylalanine, derivatized samples were measured using a GC-P-IRMS (MAT 253; Thermo Fisher Scientific, Bremen, Germany) equipped with a DB17MS 60m column with 5m pre-column (No. 122–4762; Agilent) and GC-Isolink, monitoring of ion masses 2 and 3. Standard regression curves were applied from a series of known standard enrichment values against the measured values to assess the linearity of the mass spectrometer and to account for any isotope fractionation which may have occurred during the analysis.

Plasma protein analyses

Plasma protein was extracted from basal blood samples by adding 40 μL 20% perchloric acid (PCA) to 360 μL plasma to achieve a final concentration of 2%. Samples were centrifuged at 1000 *g* at 4°C for 20 min after which the supernatant was removed. The mixed plasma protein pellet was washed with 3 washes of 2% PCA, after which the supernatant was removed. The protein pellet was hydrolyzed after adding 6 M HCl by heating at 110°C for 15–18 h. The hydrolyzed protein fraction was then dried under a nitrogen stream while being heated to 110°C. Thereafter, the hydrolyzed mixed plasma protein samples were processed via the same procedures as the muscle tissue analyses.

Western blotting

A portion of each muscle sample frozen for biochemical analyses was homogenized in seven volumes Tris buffer (20 mM Tris-HCL, 5 mM EDTA. 10 mM Na-pyrosphospate, 100 mM NaF, 2 mM Na_3VO_4, 1% Nonident P-40; pH 7.4) supplemented with protease and phosphatase inhibitors: aprotinin 10 μg mL^{-1}, leupeptin 10 μg mL^{-1}, benzamidin 3 mM and phenylmethylsulphonyl fluoride 1 mM. After homogenization, each muscle extract was centrifuged for 10 min at 10 000*g* (4°C) and sample buffer was added to the supernatant to final concentrations of 60 mM Tris, 10% glycerol, 20 mg mL^{-1} SDS, 0.1 mM dithiothreitol, 20 μg mL^{-1} bromophenol blue. The supernatant was then heated for 5 min at 100°C and immediately placed on ice. Immediately before analyses, the muscle extraction sample was warmed to 50°C and centrifuged for 1 min at 1000*g* [room temperature (RT)]. The total amount of sample loaded on the gel was based on protein content. After a Bradford assay, 30 μg protein were loaded in each lane. With the exception of mTOR, protein samples were run on a Criterion Precast TGX 4–20% gel (Order No. 567–1094; Bio-Rad) ± 90 min at 150 V (constant voltage) and transferred onto a *Trans*-blot Turbo 0.2 μm nitrocellulose membrane (Order No. 170–4159; Bio-Rad) in 7 min at 2.5 A and 25 V mTOR proteins were run and blotted for 10 min at 2.5 A and 25 V but on a Criterion Precast XT 3–8% Tris-acetate gel (Order No. 345-0130; Bio-Rad). Specific proteins were detected by overnight incubation at 4°C on a shaker with specific antibodies in 50% in PBS Odyssey blocking buffer (Part No. 927-40 000; Li-Cor Biosciences, Lincoln, NE, USA) after blocking for 60 min at RT in 50% in PBS Odyssey blocking buffer. Polyclonal primary phospho-specific antibodies, anti-phospho-mTOR (Ser^{2448}), anti-phospho-S6K1 (Thr^{389}), anti-phospho-S6K1 (Thr^{421}/Ser^{424}), anti-phospho-rpS6 (Ser^{235}/Ser^{236}), anti-phospho ACC (Ser^{79}) and anti-phospho-4E-BP1 ($Thr^{37/46}$) were purchased from Cell Signaling Technology (Danvers, MA, USA). In addition, anti-mTOR, anti-p70 S6K1, anti-RS6, anti-ACC, anti-Beclin1, anti-LC3b, anti-Atg12, anti-Myostatin and anti-4E-BP1 were also purchased from Cell Signaling Technology. Following incubation, membranes were washed three times 10 min in 0.1% PBS-Tween 20 and once for 10 min in PBS. Next, samples were incubated on a shaker (1 h at RT) with infrared secondary antibodies, donkey anti-rabbit IRDYE 800 (dilution 1:10 000; Cat. No. 611-732-127; Rockland Immunochemicals, Pottstown, PA, USA) and donkey anti-mouse IRDYE 800CW (dilution 1:10 000; Cat. No. 626–32 212; Li-Cor Biosciences) dissolved in 50% PBS Odyssey blocking buffer. After a final wash step (3 × 10 min) in 0.1% Tween 20-PBS and once 10 min in PBS, protein quantification was performed by scanning on an Odyssey Infrared Imaging System (Li-Cor Biosciences). Ponceau S staining was used to standardize for the amount of protein loaded. Phosphorylation status as a proxy of activation of the signaling proteins was expressed relative to the total amount of each protein.

mRNA analyses

Total RNA was isolated from 10 to 20 mg of frozen muscle tissue using TRIzol Reagent (Life Technologies, Invitrogen, Carlsbad, CA, USA), in accordance with the manufacturer's instructions. Total RNA quantification was carried out spectrophotometrically at 260 nm (NanoDrop ND-1000 Spectrophotometer; Thermo Fisher Scientific) and RNA purity was determined as the ratio of readings at 260/280 nm. Thereafter, first strand cDNA was synthesized from 1 μg of RNA sample using iScript cDNA synthesis kit (Bio-Rad; Cat. No. 170–8891). Taqman PCR was carried out using a 7300 Real-Time PCR System (Applied Biosystems, Foster City, CA, USA), with 2 μL of cDNA, 12.5 μL of Taqman master mix, 1.25 μL of Taqman probe and 9.25 μL of H_2O in a 25 μL final well volume. Each sample was run in duplicate, together with a serial dilution standard curve. The housekeeping gene 18S was used as an internal control. Taqman primer/probe sets were obtained from Applied Biosystems: FOXO1 (Hs 0 105 4576_m1), MuRF1 (Hs 002 61590_m1), MAFbx (Hs 0 104 1408_m1), mTOR (Hs 002 34508_m1), p70S6K (Hs 001 77357_m1), LAT1/SLC (Hs 001 85826_m1), PAT1 (Hs 0 109 2773_m1), SNAT2 (Hs 0 108 9954_m1), CD98 (Hs 003 74243_m1), cATG12 (Hs01047860_g1), LC3b (Hs00797944_s1), Beclin1 (Hs00186838_m1), Myostatin (Hs00193363_m1), PGC1-alpha (Hs01016719_m1) and 18S (Hs 0 300 3631_g1). The thermal cycling conditions used were: 2 min at 50°C, 10 min at 95°C, followed by 40 cycles at 95°C for 15 s and 60°C for 1 min. Ct values of the target genes were normalized to Ct values of the internal control and final results were calculated as relative expression against the standard curve.

Calculations

Total, exogenous, and endogenous rate of appearance ($R_{a;}$ μmol phenylalanine·kg BM^{-1}·min^{-1}) and total, exogenous, and endogenous rate of disappearance (R_d) were calculated as follows.

$$TotalR_a = \frac{F_{phe,iv} \cdot \left[pV \cdot C(t) \cdot \frac{\Delta E_{iv}}{\Delta t} \right]}{E_{phe,iv}(t)} \qquad \text{(Equation 1)}$$

$$ExoR_a = \frac{TotalR_a \cdot E_{p,oral(t)} + \left[pV \cdot C(t) \cdot \frac{\Delta E_{p,oral}}{\Delta t}\right]}{E_{oral}} \quad \text{(Equation 2)}$$

$$EndoR_a = protein\ breakdown = totalR_a - ExoR_a - F_{phe,iv} \quad \text{(Equation 3)}$$

$$Total\ Rd = totalR_a - pV \cdot \frac{\Delta C}{\Delta t} \quad \text{(Equation 4)}$$

$$ExoR_d = totalR_d \cdot \frac{E_{p,oral(t)} + \left[pV \cdot C(t) \cdot \frac{\Delta E_{p,oral}}{\Delta t}\right]}{E_{oral}} \quad \text{(Equation 5)}$$

$$EndoR_d = totalR_d - exoR_d \quad \text{(Equation 6)}$$

$F_{phe,iv}$ represents the intravenous tracer (L-[$^2H_5$]-phenylalanine) infusion rate. pV (0.125 L kg^{-1}) represents the distribution volume of phenylalanine.[57] C(t) represents the mean plasma phenylalanine concentration between two consecutive time points. $\Delta E_{iv}/\Delta t$ represents the time-dependent variation of plasma phenylalanine enrichments derived from the intravenous tracer (L-[$^2H_5$]-phenylalanine). $E_{phe,iv}(t)$ represents the mean plasma phenylalanine enrichment derived from the intravenous tracer (L-[$^2H_5$]-phenylalanine) between two consecutive time points. Exo R_a represents the rate at which dietary protein-derived phenylalanine enters the circulation. $\Delta E_{p,oral}(t)$ represents the mean plasma phenylalanine enrichment derived from the oral tracer (L-[1-^{13}C-phenylalanine]) between two consecutive time points. $\Delta E_{p,oral}/\Delta t$ represents the time dependent variation of the plasma phenylalanine enrichments derived from the oral tracer (L-[1-^{13}C-phenylalanine]). E_{oral} represents the (L-[1-^{13}C]-phenylalanine) enrichment of the dietary protein.

Phe_{plasma} represents the percentage of dietary protein-derived phenylalanine appearing in the circulation.

$$Phe_{plasma} = \frac{(AUC_{exoRa})}{Phe_{oral}} x\ BM\ x\ 100$$

AUC_{exoRa} represents the area under the curve of Exo R_a, which corresponds to the amount of dietary protein-derived phenylalanine that appeared in the circulation throughout the postprandial assessment period. Phe_{oral} represents the amount of phenylalanine ingested. BM is the participants body mass.

Whole-body protein synthesis, breakdown, oxidation, and net balance were calculated as follows:

$$Protein\ oxidation = TyrR_a \cdot \frac{E_{D4tyr}(t)}{E_{phe,iv}(t)} \cdot \frac{R_d}{F_{phe,iv} + R_d} \quad \text{(Equation 7)}$$

$$Protein\ synthesis = TotalR_d - Oxidation \quad \text{(Equation 8)}$$

$$Net\ balance = Protein\ synthesis - protein\ breakdown \quad \text{(Equation 9)}$$

Tyr R_a represents the total rate of tyrosine appearance based on L-[ring-$^2H_2$]-tyrosine infusion rate and plasma enrichments. $E_{D4\text{-}tyr(t)}$ represents the mean plasma L-[ring-$^2H_4$]-tyrosine enrichments between 2 consecutive time points. $E_{phe,iv(t)}$ represents the mean plasma L-[ring-$^2H_5$]-phenylalanine enrichment between 2 consecutive time points.

The fractional synthesis rate (FSR) of mixed-muscle, myofibrillar protein, muscle connective protein, and plasma protein was calculated using the precursor-product equation:

$$FSR(\% \cdot h^{-1}) = \frac{\Delta E_{bound}}{E_{precursor} \cdot t} \times 100\% \quad \text{(Equation 10)}$$

ΔE_{bound} is the change in plasma protein, mixed-muscle, myofibrillar, or muscle connective protein-bound L-[ring-$^2H_5$]-phenylalanine or L-[1-^{13}C]-leucine enrichments between two muscle biopsies. $E_{precursor}$ is the weighted mean plasma or muscle free L-[1-^{13}C]-leucine or L-[ring-$^2H_5$]-phenylalanine enrichment during the tracer incorporation period. *t* is the tracer incorporation time in hours.

De novo muscle protein synthesis (MPS_{denovo}) represents the amount of dietary protein-derived amino acids being incorporated into mixed-muscle protein and was calculated as follows:

$$MPS_{denovo}\,(g) = \frac{SMM \cdot SMM_{pro} \cdot \Delta E_{m-boundexo}}{E_{oral}} \quad \text{(Equation 11)}$$

SMM is the skeletal muscle mass in g. SMM_{PRO} is the protein content of skeletal muscle (20%). $\Delta E_{m\text{-}boundexo}$ is the change in muscle protein-bound L-[1-^{13}C]-phenylalanine enrichment between two muscle biopsies.

De novo plasma protein synthesis (PP_{denovo}) represents the amount of dietary protein-derived amino acids being incorporated into mixed-plasma protein and was calculated as follows:

$$PP_{denovo}\,(g) = \frac{BM \cdot BM_{blood} \cdot BLOOD_p \cdot PLASMA_{pro} \cdot \Delta E_{p-boundexo}}{E_{oral}} \quad \text{(Equation 12)}$$

BM_{blood} (0.07) is the blood fraction of body mass. $BLOOD_p$ (0.55) is the plasma fraction of blood. $PLASMA_{pro}$ (70 g L^{-1}) is the protein content of plasma. $\Delta E_{p\text{-}boundexo}$ is the change in plasma protein-bound L-[1-^{13}C]-phenylalanine between two plasma samples.

The contribution of exogenous and endogenous amino acids to myofibrillar protein synthesis was calculated as follows:

$$FSR\ from\ exogenous\ AA(\%) = \frac{\Delta E_{m-boundexo} / E_{oral}}{\Delta E_{m-bound-iv} / E_{phe.iv}} \cdot 100 \quad \text{(Equation 13)}$$

$$FSR\ from\ endogenous\ AA(\%) = 100 - FSR\ from\ exogenous\ AA \quad \text{(Equation 14)}$$

$\Delta E_{m\text{-}boundexo}$ and $\Delta E_{m\text{-}bound\text{-}iv}$ are the changes in myofibrillar protein-bound L-[1-^{13}C]-phenylalanine and L-[$^2H_5$]-phenylalanine between biopsies.

QUANTIFICATION AND STATISTICAL ANALYSIS

All data in text are expressed as mean ± SD. Data in box and whiskers include the median (line), mean (cross), interquartile range (box) and minimum and maximum values (tails). Time-dependent variables were analyzed by a two-factor repeated-measures ANOVA with time as a within-subject factor and treatment as a between-subjects factor. Time-independent variables were analyzed by a one-way ANOVA with treatment as between-subject factor. All analysis was carried out for the period starting at the time of experimental drink administration ($t = 0$ min). The pre-registered primary analysis was myofibrillar protein synthesis rate over the 0–720 min period following administration of the experimental drinks. In case of a significant effect, a Bonferroni post hoc test was applied to locate group differences. All calculations were performed using SPSS version 27.0 (IBM Corp). Statistical significance was set at $p < 0.05$.

ADDITIONAL RESOURCES

The trial was prospectively registered at the Netherlands Trial Register (NL7700) and the clinical trials were conducted between June 2019 and March 2020 at Maastricht University in Maastricht, the Netherlands. This study was approved by the Medical Research Ethics Committee Academic Hospotal Maastricht/Maastricht University, the Netherlands (METC 19-012) and was performed in accordance with the Helsinki Declaration of 1975 as revised in October 2013. All participants were informed about the purpose of the study, the experimental procedures, and possible risks before providing written informed consent to participate. The study was independently monitored by the Clinical Trial Center Maastricht.

Cell Reports Medicine, Volume *4*

Supplemental information

The anabolic response to protein ingestion during recovery from exercise has no upper limit in magnitude and duration *in vivo* in humans

Jorn Trommelen, Glenn A.A. van Lieshout, Jean Nyakayiru, Andrew M. Holwerda, Joey S.J. Smeets, Floris K. Hendriks, Janneau M.X. van Kranenburg, Antoine H. Zorenc, Joan M. Senden, Joy P.B. Goessens, Annemie P. Gijsen, and Luc J.C. van Loon

SUPPLEMENTAL TITLES AND LEGENDS

Supplemental Figure 1. Plasma amino acid responses following protein ingestion. Related to figure 1.

Supplemental Figure 2. Muscle free amino acid response following protein ingestion. Related to figure 3.

Supplemental table 1. Subject's characteristics. Related to figure 1.

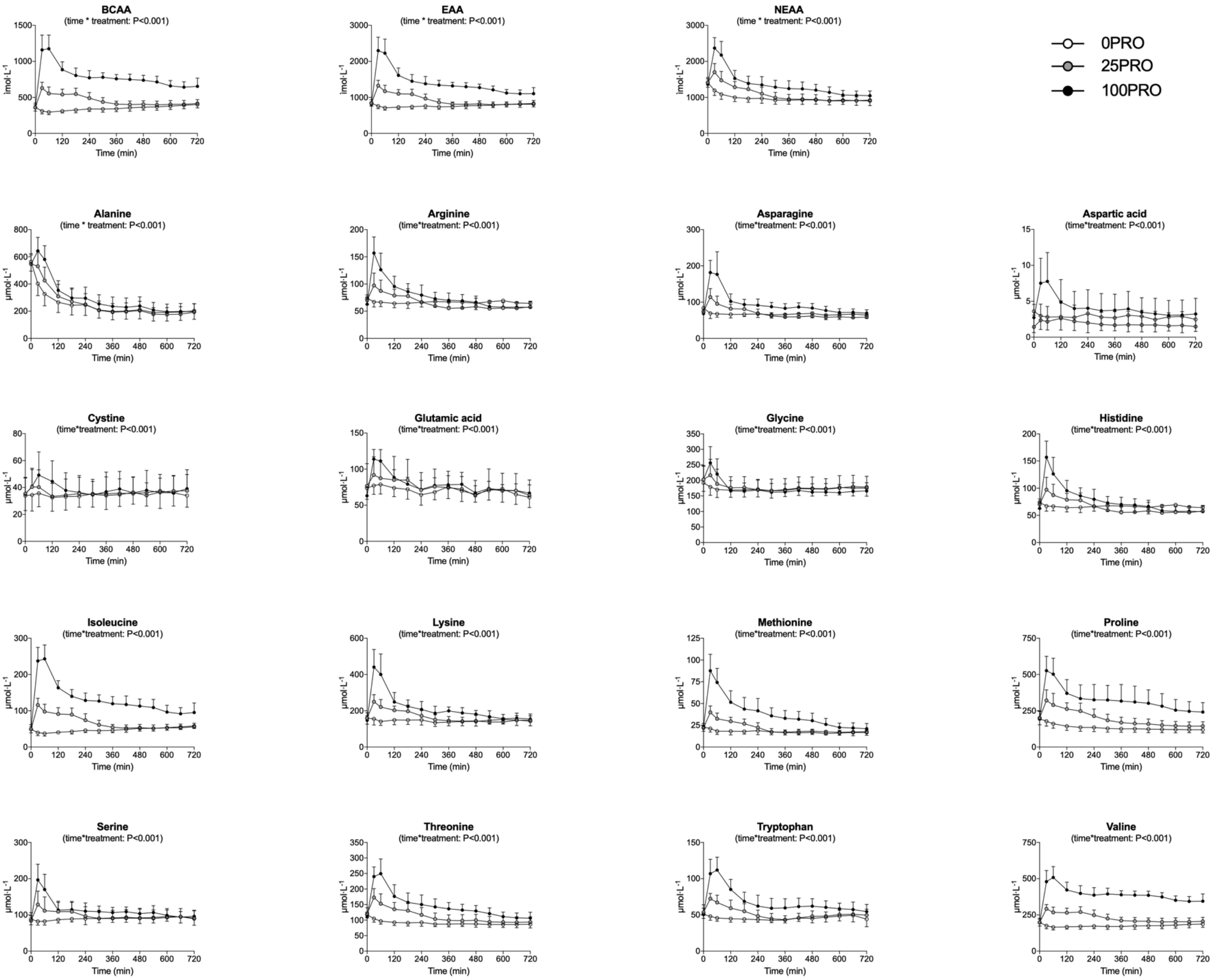
0PRO
25PRO
100PRO
BCAA
(time * treatment: P<0.001)
EAA
(time * treatment: P<0.001)
NEAA
(time * treatment: P<0.001)
Alanine
(time * treatment: P<0.001)
Arginine
(time*treatment: P<0.001)
Asparagine
(time*treatment: P<0.001)
Aspartic acid
(time*treatment: P<0.001)
Cystine
(time*treatment: P<0.001)
Glutamic acid
(time*treatment: P<0.001)
Glycine
(time*treatment: P<0.001)
Histidine
(time*treatment: P<0.001)
Isoleucine
(time*treatment: P<0.001)
Lysine
(time*treatment: P<0.001)
Methionine
(time*treatment: P<0.001)
Proline
(time*treatment: P<0.001)
Serine
(time*treatment: P<0.001)
Threonine
(time*treatment: P<0.001)
Tryptophan
(time*treatment: P<0.001)
Valine
(time*treatment: P<0.001)
µmol·L-1
Time (min)
0 120 240 360 480 600 720

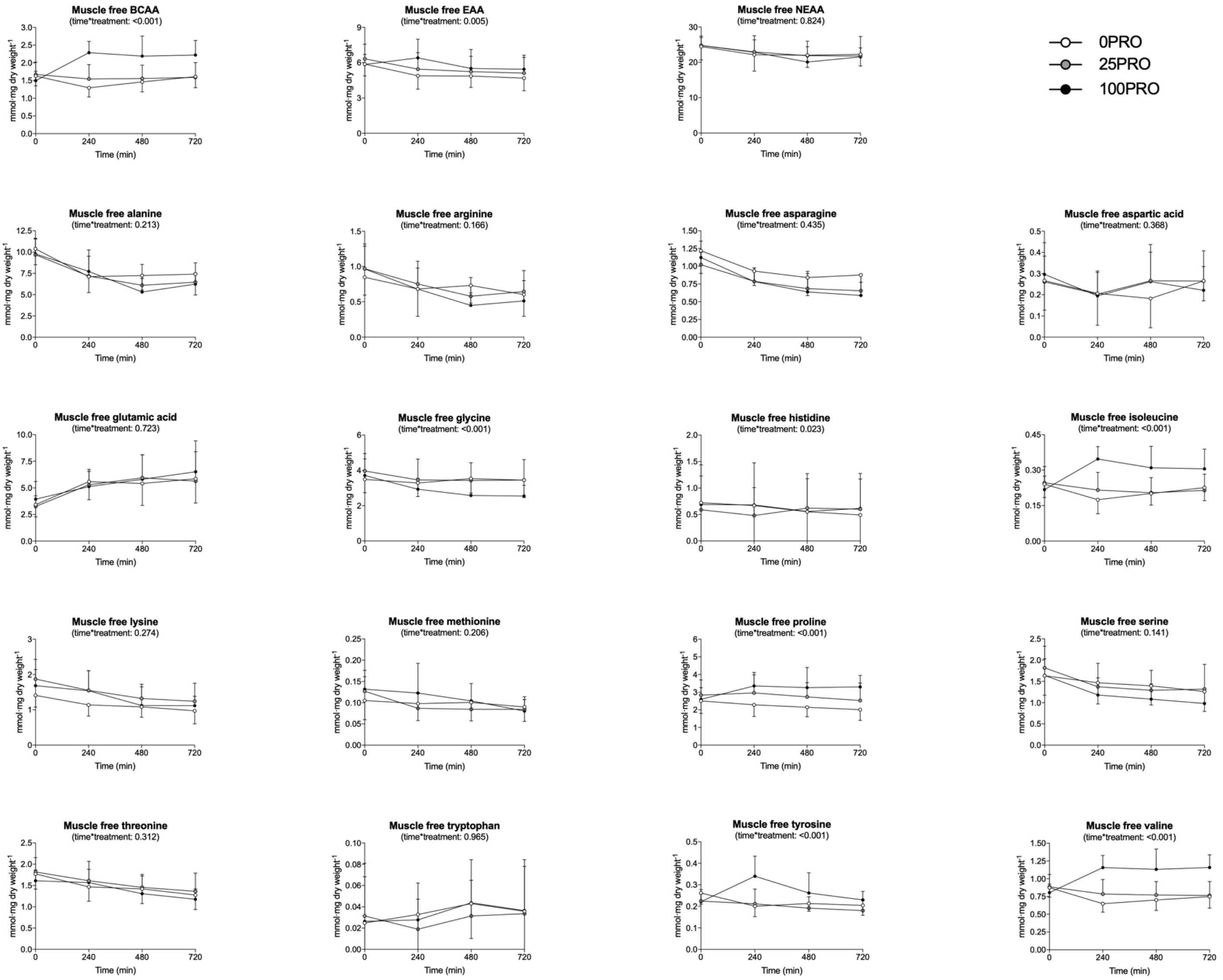
Muscle free BCAA
(time*treatment: <0.001)
Muscle free EAA
(time*treatment: 0.005)
Muscle free NEAA
(time*treatment: 0.824)
0PRO
25PRO
100PRO
Muscle free alanine
(time*treatment: 0.213)
Muscle free arginine
(time*treatment: 0.166)
Muscle free asparagine
(time*treatment: 0.435)
Muscle free aspartic acid
(time*treatment: 0.368)
Muscle free glutamic acid
(time*treatment: 0.723)
Muscle free glycine
(time*treatment: <0.001)
Muscle free histidine
(time*treatment: 0.023)
Muscle free isoleucine
(time*treatment: <0.001)
Muscle free lysine
(time*treatment: 0.274)
Muscle free methionine
(time*treatment: 0.206)
Muscle free proline
(time*treatment: <0.001)
Muscle free serine
(time*treatment: 0.141)
Muscle free threonine
(time*treatment: 0.312)
Muscle free tryptophan
(time*treatment: 0.965)
Muscle free tyrosine
(time*treatment: <0.001)
Muscle free valine
(time*treatment: <0.001)
mmol·mg dry weight-1
Time (min)

Supplemental table 1. Subject's characteristics

	**0PRO** (n=12)	**25PRO** (n=12)	**100PRO** (n=12)	**Total** (n=36)
Age (y)	24 ± 5	25 ± 5	27 ± 7	25 ± 6
Body mass (kg)	75.5 ± 14.1	79.1 ± 10.4	75.7 ± 10.6	76.8 ± 11.6
Height (m)	1.79 ± 0.08	1.83 ± 0.07	1.81 ± 0.08	1.81 ± 0.08
BMI ($kg \cdot m^{-2}$)	23.5 ± 3.2	23.6 ± 3.2	23.0 ± 2.5	23.4 ± 2.9
Body fat (%)	17 ± 6	16 ± 6	14 ± 4	16 ± 5
Fat free mass (kg)	62.5 ± 7.7	66.3 ± 7.5	64.6 ± 8.1	64.5 ± 7.7
Skeletal muscle mass (kg)	32.3 ± 4.1	34.3 ± 4.4	33.4 ± 4.6	33.4 ± 4.3
1RM Leg Press (kg)	215 ± 61	197 ± 44	204 ± 39	205 ± 48
1RM Leg Extension (kg)	107 ± 28	108 ± 22	114 ± 24	110 ± 25
1RM Lat pulldown (kg)	80 ± 24	79 ± 14	79 ± 15	79 ± 18
1RM Chest press (kg)	89 ± 33	90 ± 23	93 ± 21	91 ± 25
Energy intake ($MJ \cdot kg\ BM^{-1} \cdot d^{-1}$)	0.13 ± 0.02	0.12 ± 0.03	0.14 ± 0.03	0.13 ± 0.03
Protein intake ($g \cdot kg\ BM^{-1} \cdot d^{-1}$)	1.4 ± 0.4	1.1 ± 0.3	1.5 ± 0.3	1.3 ± 0.3
Carbohydrate intake ($g \cdot kg\ BM^{-1} \cdot d^{-1}$)	3.5 ± 0.9	3.5 ± 0.9	4.0 ± 1.3	3.7 ± 1.1
Fat intake ($g \cdot kg\ BM^{-1} \cdot d^{-1}$)	1.1 ± 0.3	1.1 ± 0.3	1.2 ± 0.4	1.1 ± 0.3

Values represent mean ± SD. Dietary records data in PLA: n=11.
